# Two rice cultivars recruit different rhizospheric bacteria to promote aboveground regrowth after mechanical defoliation

**DOI:** 10.1128/spectrum.01254-24

**Published:** 2024-12-09

**Authors:** Changjin Jiang, Fei Wang, Jinling Tian, Wanyuan Zhang, Kabin Xie

**Affiliations:** 1 National Key Laboratory of Crop Genetic Improvement, Hubei Hongshan Laboratory, Huazhong Agricultural University, Wuhan, China; 2 Hubei Key Laboratory of Plant Pathology, Huazhong Agricultural University, Wuhan, China; 3 Shenzhen Institute of Nutrition and Health, Huazhong Agricultural University, Wuhan, China; 4 Shenzhen Branch, Guangdong Laboratory for Lingnan Modern Agriculture, Genome Analysis Laboratory of the Ministry of Agriculture, Agricultural Genomics Institute at Shenzhen, Chinese Academy of Agricultural Sciences, Shenzhen, China; College of Life Sciences, Nanchang University, Nanchang, Jiangxi, China

**Keywords:** bacterial microbiome, mechanical defoliation, rice, regrowth promotion, *Rhodocyclaceae*

## Abstract

Plants have evolved the ability to regrow after mechanical defoliation and environmental stresses. However, it is unclear whether and how defoliated plants exploit beneficial microbiota from the soil to promote aboveground regrowth. Here, we compared the defoliation-triggered changes in the root exudation and bacterial microbiome of two rice cultivars (*Oryza sativa* L *ssp*.), *indica/xian* cultivar Minghui63 and *japonica/geng* cultivar Nipponbare. The results show that reciprocal growth promotion existed between defoliated Minghui63 seedlings and soil bacteria. After the leaves were removed, the Minghui63 seedlings displayed approximately 1.5- and 2.1-fold higher root exudation and leaf regrowth rates, respectively, than did the Nipponbare seedlings. In field trials, Minghui63 and Nipponbare enriched taxonomically and functionally distinct bacteria in the rhizosphere and root. In particular, Minghui63 rhizosphere and root communities depleted bacteria whose functions are related to xenobiotics biodegradation and metabolism. The microbiome data implied that the bacterial family *Rhodocyclaceae* was specifically enriched during the regrowth of defoliated Minghui63 rice. We further isolated a *Rhodocyclaceae* strain, *Uliginosibacterium gangwonense* MDD1, from rice root. Compared with germ-free conditions, MDD1 inoculation promoted the aboveground regrowth of defoliated Minghui63 by 61% but had a weaker effect on Nipponbare plants, suggesting cultivar-specific associations between regrowth-promoting bacteria and rice. This study provides novel insight into microbiota‒root‒shoot communication, which is implicated in the belowground microbiome and aboveground regrowth in defoliated rice. These data will be helpful for microbiome engineering to increase rice resilience to defoliation and environmental stresses.

**IMPORTANCE:**

As sessile organisms, plants face a multitude of abiotic and biotic stresses which often result in defoliation. To survive, plants have evolved the ability to regrow leaves after stresses and wounding. Previous studies revealed that the rhizosphere microbiome affected plant growth and stress resilience; however, how belowground microbiota modulates the aboveground shoot regrowth is unclear. To address this question, we used rice, an important crop worldwide, to analyze the role of rhizosphere microbiota in leaf regrowth after defoliation. Our data indicate mutual growth promotion between defoliated rice and rhizosphere bacteria and such beneficial effect is cultivar specific. The microbiome analysis also led us to find a *Uliginosibacterium gangwonense* strain that promoted rice cv. MH63 leaf regrowth. Our findings therefore present a novel insight into plant-microbiome function and provide beneficial strains that potentially enhance rice stress resilience.

## INTRODUCTION

Enormous microorganisms inhabit the rhizosphere, endosphere, and phyllosphere of plants. These plant-associated microbes, including their genetic elements, metabolites, and interactions, constitute the plant microbiome, which is crucial for plant growth, health, and adaptation (1–3). Except for a few bacterial taxa inherited from seeds (4), most rhizospheric and endophytic bacteria come from indigenous soil through the active selection of plant hosts (5, 6). The rhizosphere and endosphere microbiota of crops are determined by soil physiochemical properties (7), plant genotypes and developmental stages (8), abiotic and biotic stresses (3), and cropping practices (9). The bacterial microbiome plays a critical role in plant disease resistance (10, 11), development (12), nutrient acquisition and uptake (13, 14), and stress tolerance (15). Hence, engineering crop microbiomes with beneficial functions is a promising strategy for increasing agricultural productivity (16, 17).

Upon pathogen and insect attack, plants change their microbiomes to defend against pathogen invasion (18). A classic example is disease-suppressive soil, in which beneficial bacteria were enriched after growing disease-resistant cultivars, and the bacterial legacies benefited subsequent generations by suppressing soil-borne pathogens such as *Rhizoctonia solani* (19, 20) and *Fusarium* (21, 22). Bacteria in seeds (11), leaves (23), and flowers (24) were also exploited by plant hosts to suppress pathogens. These findings, particularly studies on disease-suppressive soils, lead to a cry-for-help model to explain the molecular and ecological mechanisms by which plants exploit bacterial consortia to cope with biotic stresses (25, 26). In the cry-for-help model, root exudates, which can be used by bacteria for energy and biomass production, are recognized as a critical factor for enriching beneficial bacteria in the rhizosphere and root endosphere (27–29). Plants exude 5%–21% of photosynthetically fixed carbon through roots (30). Root exudates include diverse metabolites, and their composition is affected by host genetics, soil physiochemical properties, and stresses (30, 31). A recent study in *Arabidopsis* revealed that reducing plant photosynthesis impacted root exudates and triggered belowground changes in the microbiome, suggesting that a microbiome‒root‒shoot circuit regulates aboveground stress responses in plants (32).

As sessile organisms, plants not only have evolved the ability to tolerate stress but also have evolved the ability to regrow after leaf wilting and mechanical wounding. Defoliation, the removal of leaves by disease, insect feeding, the application of defoliants, mechanical injury, and the harvesting of grains, is a severe wounding for crops. Perennial plants are autonomously defoliated in the cold season and regrow in the next year. In addition, artificial defoliation is a common cropping technique in agriculture. However, our knowledge about the functions of the microbiome in plant regrowth after defoliation is limited. Since root exudates are mainly from photosynthetic products, a drastic reduction in photosynthesis in defoliated plants caused source‒sink conversion (33, 34), thereby altering root physiology. For example, carbon exudation was induced after defoliation in a temperate grassland (35). The defoliation intensity of grasses also shifted the soil microbiota in northern mixed-grass prairies (36). Using biosensors and agar systems, M. S. Thilakarathna and M. N. Raizada (37) found that defoliation triggered the rapid release of glutamine from young roots of forage legumes. Rice (*Oryza sativa* L. *ssp*.) is one of the most important staple crop species and a model system for monocot plants. Rice suffers defoliation by disease, insect feeding, and crop management. Defoliated rice can rapidly regrow from stubbles. In particular, ratooning rice, which regrows from rice stubbles after grain harvesting, is a popular cropping strategy in many areas for increasing rice production (38). The regrowth ability of defoliated rice is regulated by host genetics, environment, and crop management (39). However, the effect of the microbiome on rice regrowth after defoliation has not been explored.

Unlike other environmental stresses (e.g., drought and high salinity), which directly impact both plant hosts and soil biota, defoliation has no direct effect on soil microbiota but affects rhizospheric bacteria through plant hosts. We hypothesized that defoliation treatment can be used to perturb plant–microbiota interactions. Therefore, profiling the microbiome during the regrowth of defoliated plants would provide novel insight into beneficial interactions between bacterial communities and plants. We aimed to characterize the associations between the bacterial microbiome and defoliated rice plants using two cultivated varieties, Minghui63 (MH63) and Nipponbare (NIP). MH63 is an elite variety of *indica* rice (*Oryza sativa* L. ssp. *indica/xian*) and is widely used in rice breeding in China, while NIP is a *japonica* subspecies (*Oryza sativa* L. ssp. *japonica/geng*) and is the reference variety for rice genomics. More importantly, MH63 and NIP vary in many agronomic traits and microbiota compositions (40–42). In this study, we combined the 16S rRNA gene amplicon sequencing and co-cultivation assays to analyze the effects of microbiota on leaf regrowth of MH63 and NIP rice. We found that rice enriched beneficial bacteria for regrowth upon defoliation in a variety-specific manner. The microbiome analysis led to the discovery of the *Rhodocyclaceae* bacterial isolate *Uliginosibacterium gangwonense* MDD1, which can promote MH63 regrowth after defoliation. This study provides novel insights into plant–microbiome interactions during plant regrowth after mechanical injury, which could be helpful for microbiome engineering to increase rice resilience to mechanical injury and stress.

## RESULTS

### Reciprocal growth promotion between defoliated MH63 rice and root bacteria

**Fig 1:**
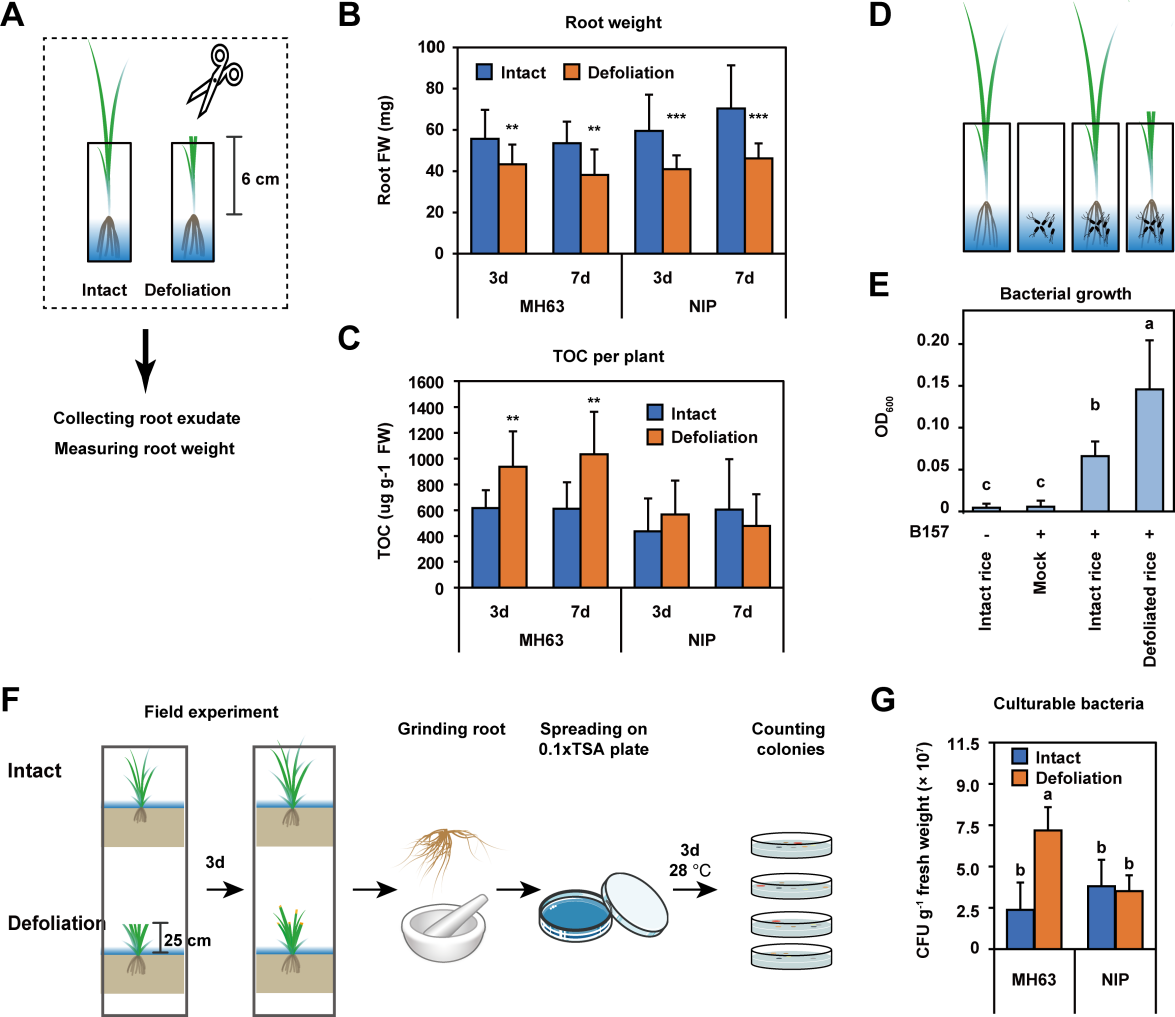
Defoliating MH63 rice increased root exudates and promoted bacterial growth. (**A**) Diagram of defoliation treatment of rice seedlings in sterile hydroponic solution. (**B and C**) Comparison of the root fresh weight and exudates of intact and defoliated rice seedlings at 3 and 7 days after defoliation. MH63, Minghui 63. NIP, Nipponbare. TOC, total organic carbon in rice exudates. FW, fresh weight. **P < 0.01, ***P < 0.001, Student’s *t* test (*n* = 11–17). (**D and E**) Growth of *S. marcescens* strain B157 after cocultivation with intact and defoliated MH63 seedlings in MS media. Intact and defoliated rice seedlings were inoculated with (+) or without (−) B157 strains. The optical density (OD) at 600 nm of MS media was measured after 3 days of cocultivation. Mock, MS media inoculated with B157 only. The letters in the plot indicate significant differences (one-way ANOVA followed by post hoc Tukey’s HSD, *P* < 0.01, *n* = 8). (**F and G**) Comparisons of number of culturable bacteria associated with intact and defoliated roots of two cultivars. The letters in the plot indicate significant differences (one-way ANOVA followed by post hoc Tukey’s HSD, *P* < 0.01, *n* = 5). CFU, colony forming unit.

To examine the effect of defoliation on rice, the fresh weight and exudates of roots were examined in MH63 and NIP seedlings. The seedlings of the two cultivars were grown in a sterilized hydroponic solution, and defoliation treatment was performed by cutting 2/3 of the shoots, which removed all leaf blades (Fig. 1A). Compared to that of the intact rice seedlings, defoliation significantly reduced the fresh weight of roots of both cultivars (Student’s *t* test, *P* < 0.01; Fig. 1B). The root weights of MH63 seedlings decreased by 22% and 28.9% at 3 and 7 days after defoliation, respectively. Similar reductions in root weights were observed for the defoliated NIP seedlings. The root exudates were estimated by measuring the total organic carbon (TOC). Interestingly, on average, defoliated MH63 roots exuded 1.5-fold more TOC than did those of intact plants. By contrast, a comparable amount of TOC was exuded by defoliated and intact NIP rice plants (Fig. 1C).

We next explored whether defoliation treatment affected the growth of beneficial bacteria. To this end, the *Serratia marcescens* strain B157, which was isolated from the rice rhizosphere in this study, was cocultured with MH63 seedlings in 1/2-strength Murashige and Skoog (MS) media without sucrose (Fig. 1D). *S. marcescens* is widely present in water (43) and is considered a rice growth-promoting bacterium (44). B157 is close to the entomopathogenic bacterium *S. marcescens* S-JS1 (Fig. S1 and S2), which can increase rice growth and defense against *Nilaparvata lugens* (45). More importantly, B157 did not grow in liquid MS media unless it was cocultured with rice seedlings (Fig. 1E), rendering this strain an indicator bacterium to measure rice exudates. The *S. marcescens* B157 cocultured with rice in MS media and its growth was assessed by measuring the optical density at 600 nm. Compared with that of intact rice plants, the growth of *S. marcescens* B157 increased twofold when cocultivated with defoliated rice seedlings (one-way ANOVA followed by post hoc Tukey’s HSD test, *P* < 0.01; Fig. 1E). These results confirm that increased root exudates from defoliated MH63 plants promote the growth of *S. marcescens* B157.

To further confirm that defoliation stimulates the growth of root bacteria, we defoliated 2-month-old plants in the field and counted the culturable bacteria after 3 days (Fig. 1F). The results show that bacterial load of the defoliated MH63 root was approximately three times greater than that of intact plants (Fig. 1G). Consistent with the TOC measurement results (Fig. 1C), defoliation had no effect on the NIP root (Fig. 1G). These results indicate that defoliated MH63 rice promoted the growth of root bacteria.

**Fig 2:**
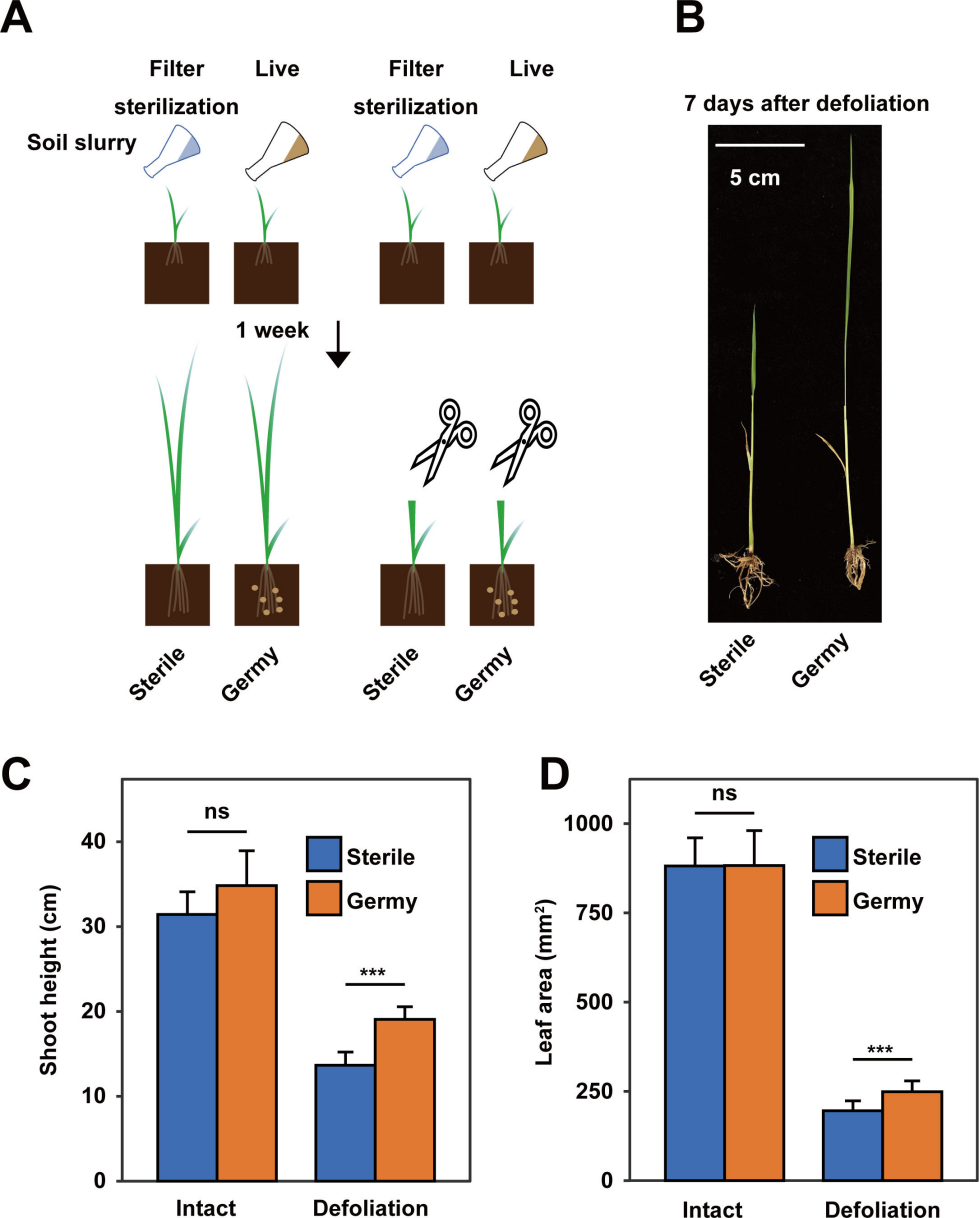
Soil microbial communities promote the regrowth of MH63 seedlings after defoliation treatment. (**A**) Diagram of the experimental design for defoliation treatment of MH63 rice seedlings grown in sterile and germy soil. Live and sterilized soil slurry prepared from paddy fields was used to inoculate intact and defoliated rice seedlings. (**B**) Photograph of MH63 seedlings at 7 days after defoliation treatment. (**C and D**) Comparisons of shoot height and leaf area of intact and defoliated rice seedlings at 7 days after defoliation in sterile and germy soils. *** and ** indicate *P* < 0.001 and 0.01, respectively; ns, no significant difference (Student’s *t* test, *n* = 8).

To explore the role of the microbiota in the regrowth of aboveground parts of defoliated plants, we compared the plant height and leaf area of defoliated MH63 seedlings growing in sterilized and germy soil. In this experiment, 1-week-old axenic rice seedlings were inoculated with soil slurry from MH63 roots growing in a paddy field (germy condition); the control groups were inoculated with the filter-sterilized soil slurry (sterile condition) (Fig. 2A). We observed that the defoliated rice plants grew slower under sterile conditions than in germy conditions. On the 7th day after cutting the shoots, the shoot height of defoliated rice seedlings under germy conditions was 28.3% higher than that under sterile conditions (Fig. 2B and C). The leaf area of defoliated plants in germy soil was 21.4% greater than that in sterile soil (Student’s *t* test, *P* < 0.01; Fig. 2D). In the same experiment, the growth of intact rice plants did not significantly differ between sterile and germy soil conditions (Fig. 2C and D).

### Defoliation affected the diversities of the rice rhizosphere and root microbiota in paddy fields

**Fig 3:**
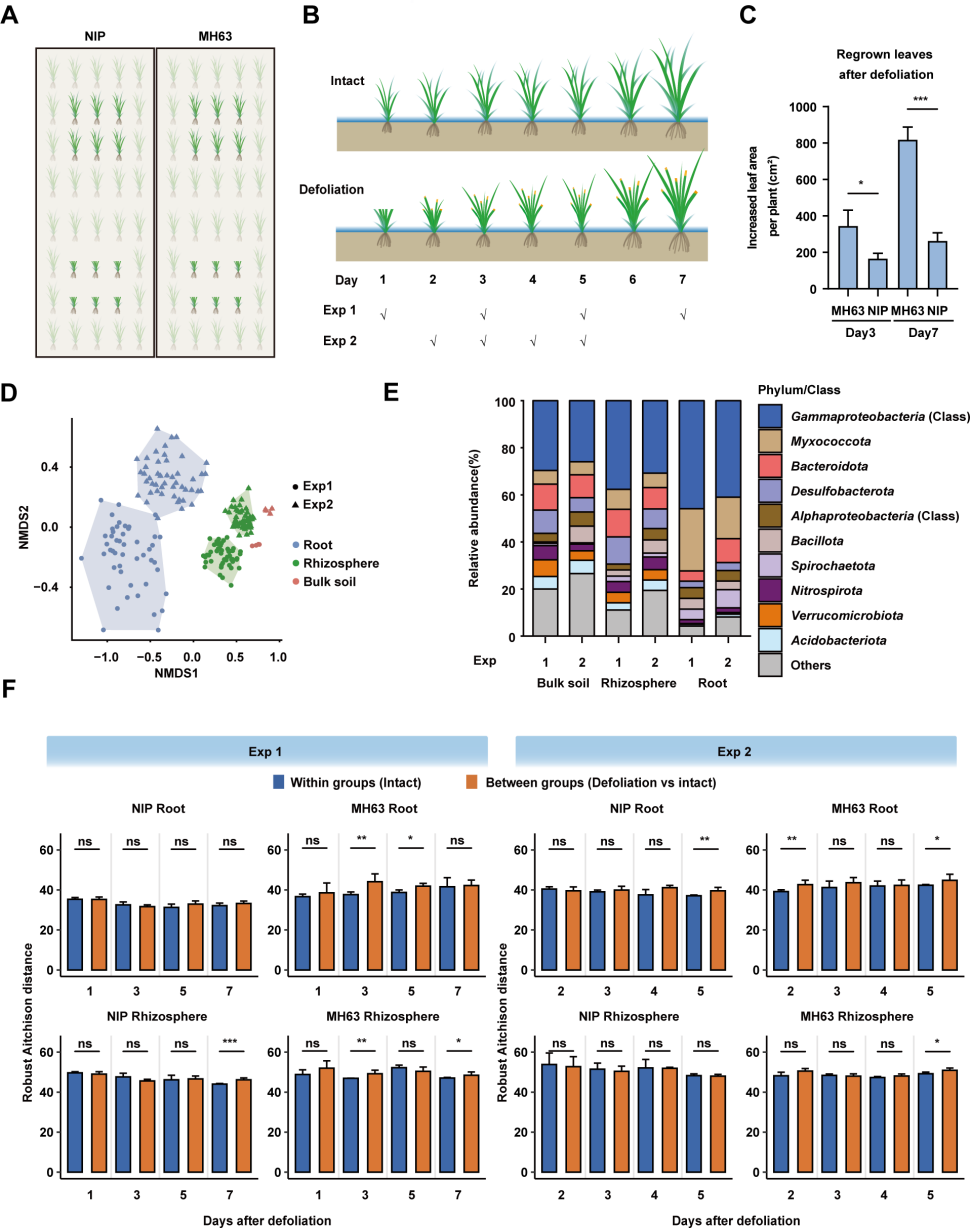
Comparisons of root and rhizosphere bacterial diversities after defoliation treatments in paddy fields. (**A and B**) Schematics showing the planting pattern in the field, defoliation treatment, and sampling timepoints in the two experiments. (**C**) Comparisons of the area of regrown leaves at 3 and 7 days after defoliation. All leaf blades were removed in the defoliation treatment (see also Supporting Information Fig. S3). * and ***, *P* < 0.05 and 0.001 (Student’s *t* test, *n* = 3). (**D**) Nonmetric multidimensional scaling (NMDS) ordination plots (first two dimensions) of the bacterial communities of intact and defoliated rice plants in two experiments. (**E**) Comparisons of the relative abundances of bacterial phyla in the two experiments. The updated bacterial phyla names from Oren and Garrity (46) are used, including *Bacillota* (formerly known as *Firmicutes*) and *Pseudomonadota* (formerly known as *Proteobacteria*). *Pseudomonadota* (aka. *Proteobacteria*), including *Alphaproteobacteria* and *Gammaproteobacteria*, are shown at class level. Others, bacterial phyla with relative abundances less than 1%. (**F**) Pairwise comparisons of Robust Aitchison distances between groups (defoliated vs intact) and within groups (intact rice plants). Error bar, standard deviation; ns, no significant difference; * and ** indicate *P* < 0.05 and 0.01, respectively (Student’s *t* test).

The reciprocal growth-promoting effect between defoliated rice and soil bacteria prompted us to examine the changes in rice root microbiomes after defoliation in paddy fields. To this end, we sampled the rhizosphere and root (including rhizoplane and endosphere compartments) bacterial communities from defoliated and intact rice plants in two consecutive years (2018 and 2019, referred to as Exp1 and Exp2, respectively) in the same field (Fig. 3A and B; Fig. S3). In both experiments, the defoliation treatment was performed at the tillering stage (6 weeks after transplanting), and new leaves were rapidly regrown from defoliated stubbles of the two cultivars under field conditions (Fig. S3). Notably, the area of regrown leaves of MH63 was 2 and 4 times greater than that of NIP at 3 and 7 days after defoliation (Fig. 3C), respectively, indicating that MH63 also has a higher regrowth ability than NIP under field conditions.

The rhizosphere and root bacterial communities of MH63 and NIP were compared at four time points within 1–7 days after defoliation (Fig. 3B). The day 1 samples in Exp2 were not available due to contamination. The bulk soils (four replicates) were also sampled at the same time to monitor the variations in soil microbiota between the 2 years. To analyze the bacterial community composition, the ZOTU pipeline, which has comparable accuracy to amplicon sequence variants (47), was used to denoise 16S rRNA gene amplicon reads (48–50). After quality filtering, a total of 14,315,485 high-quality reads were obtained from 200 samples and denoised into 16,559 bacterial ZOTUs (Table S1). The rarefaction curves suggest that the sequencing depth was sufficient to capture the bacterial diversities in both experiments (Fig. S4). The ZOTU counts were rarefied to 15,033 reads per sample to compare the bacterial diversities. NMDS ordinations showed that the assemblages of bacterial communities were distinct between the two experiments and three compartments (root, rhizosphere, and bulk soil) (Fig. 3D). The relative abundances of bacterial phyla significantly varied among the bulk soil, rhizosphere, and root samples from Exp1 and Exp2 (Fig. 3E). Defoliation had significant effects on the Shannon indices in Exp1 (Fig. S5; linear mixed model, *P* < 0.05) but had no effect on the α diversity in Exp2. For β diversity indices, we tested whether the Robust Aitchison distances between groups (defoliated vs intact) were higher than those of within-group samples (intact plant samples). Interestingly, the Robust Aitchison distances increased at 1–2 timepoints after defoliation for both cultivars (Student’s *t* test, *P* < 0.05; Fig. 3F), except for the NIP root samples in Exp1 and rhizosphere samples in Exp2. These results indicated that defoliation transiently perturbed the diversity of the bacterial communities of the rice plants in the paddy field.

We used PERMANOVA (51) to test the significance of the effects of different factors (variety, sampling timepoint, and defoliation) on bacterial community composition in the two experiments. Rice variety and sampling timepoint significantly affected the β diversity of the rhizosphere and root bacterial microbiota (variety: *R^2^* = 0.071–0.210, sample timepoint: *R^2^* = 0.099–0.183, all *P* < 0.01; Table 1). Except for the rhizosphere communities in Exp2, defoliation also significantly affected the bacterial composition of these communities (*R^2^* = 0.024–0.038, all *P* < 0.05; Table 1) but explained less variance than did variety and sampling timepoint. For the rhizosphere communities of Exp2, the sampling timepoint (*R^2^* = 0.183, *P* < 0.01) explained more of the variance than did the variety (*R^2^* = 0.071, *P* < 0.01), and the effect of defoliation was not statistically significant (*R^2^* = 0.020, *P* = 0.103). These results suggested that rice variety and sampling timepoint dominantly shaped the assemblages of bacterial communities; however, defoliation contributed to approximately 2% of the variance in the rhizospheric and root bacterial communities of rice.

**TABLE 1:** The effects of variety, sampling timepoint, and defoliation on bacterial communities in two experiments^*a*^

Factors	Root		Rhizosphere	
	R^2^	*P* value	R^2^	*P* value
**Exp1 (2018**)				
Variety (V)	0.210	0.001**	0.146	0.001**
Timepoint (T)	0.118	0.001**	0.105	0.001**
Defoliation (D)	0.024	0.034*	0.038	0.004**
V × T	0.108	0.001**	0.075	0.009**
V × D	0.031	0.006**	0.026	0.035*
T × D	0.066	0.011*	0.057	*0.096*
V × T × D	0.077	0.003**	0.054	*0.152*
**Exp2 (2019**)				
Variety (V)	0.204	0.001**	0.071	0.001**
Timepoint (T)	0.099	0.002**	0.183	0.001**
Defoliation (D)	0.027	0.044*	0.020	*0.103*
V × T	0.065	*0.061*	0.097	0.001**
V × D	0.019	*0.205*	0.018	*0.197*
T × D	0.054	*0.176*	0.061	0.031*
V × T × D	0.057	*0.121*	0.055	*0.121*

^*a*^ PERMANOVA was used to test the contributions of different factors on variance of bacterial communities. Bold numbers indicate statistical significance (α = 0.05). * indicates *P* < 0.05; ** indicates *P* < 0.01.

### Distinct bacterial taxa were enriched and depleted in the two rice varieties after defoliation

**Fig 4:**
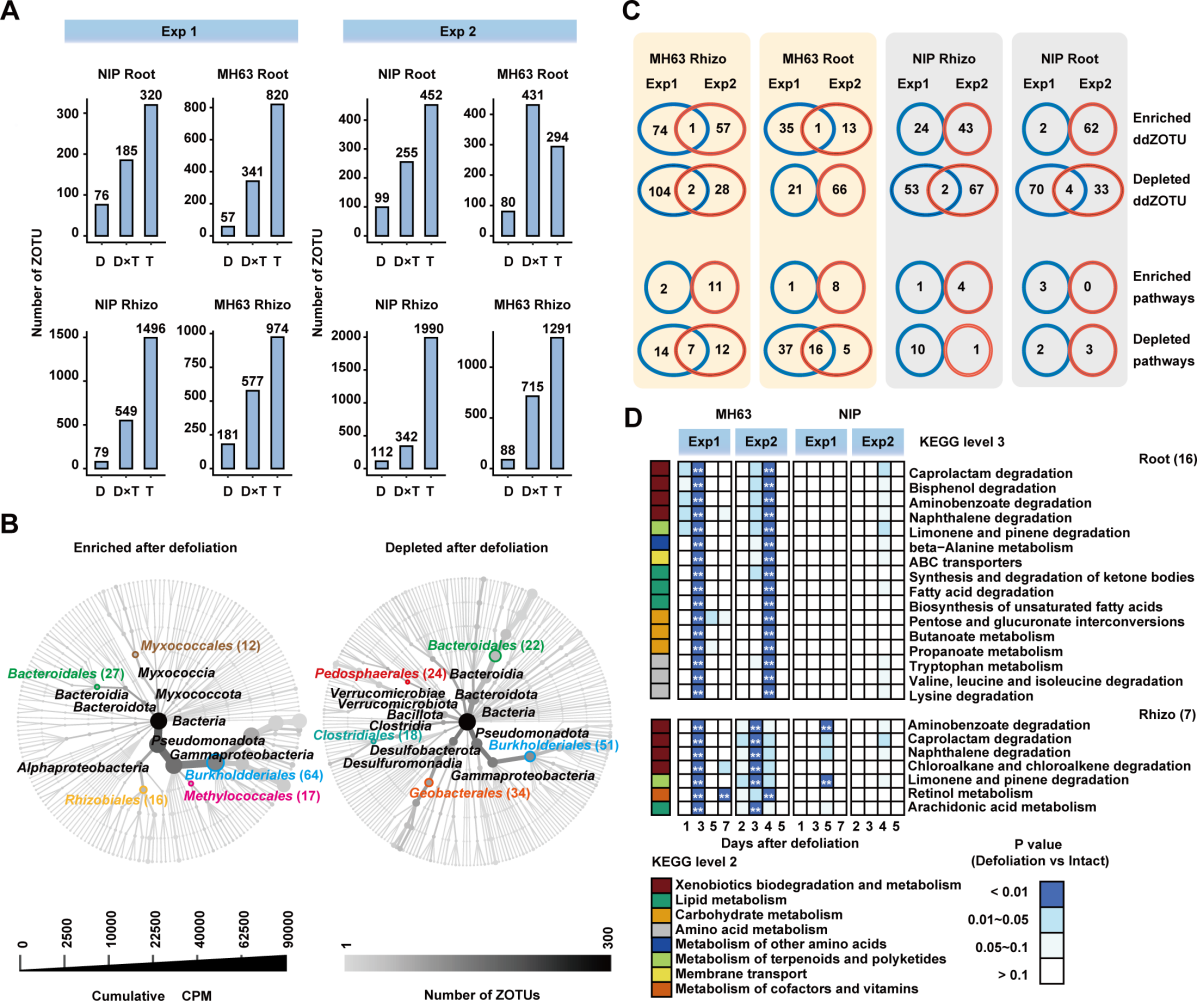
Bacterial taxa associated with defoliation in two cultivars. (**A**) Number of differential ZOTUs affected by defoliation treatment (**D**), sampling timepoint (**T**), and their interactions (D × T) (see also Supporting Information Table S2). (**B**) Phylogenetic tree of ddZOTUs. *Pseudomonadota* (formerly known as *Proteobacteria*) and *Bacillota* (formerly known as *Firmicutes*) are new phyla names from Oren and Garrity (46). The nodes in the outermost cycle represent 676 ddZOTUs. Line width indicates cumulative CPM (count per million), and the gray intensities of lines/points indicate the number of ZOTUs at different taxonomic levels. The number in parentheses indicates the number of ddZOTUs belonging to the corresponding bacterial orders. (**C**) Venn diagrams showing ddZOTUs and predicted KEGG pathways consistently detected in Exp1 and Exp2. Of note, only 1 ZOTU (ZOTU849) was consistently enriched in both the MH63 root and rhizosphere communities (see also Table 2). (**D**) Heatmap showing the pathways that were consistently depleted in both Exp1 and Exp2. The sidebar color denotes different catalogs of KEGG pathways (level 2). The number of KEGG pathways (level 3) is shown in parentheses. ** indicate *P* values < 0.01, respectively (Weltch’s t test, *n* = 3).

We then examined which bacterial taxa changed upon defoliation. Considering the vast variations in soil bacterial communities in the two experiments (Fig. 3D; Fig. S6), the differential ZOTUs of Exp1 and Exp2 were separately analyzed using Analysis of Compositions of Microbiomes with Bias Correction 2 (ANCOM-BC2). A total of 676 ZOTUs (referred to as ddZOTUs) displayed significant changes after defoliation (fold change >1.5, *P* < 0.01; Table S2). A comparative number of ddZOTUs was obtained for the MH63 and NIP samples in the root and rhizosphere compartments in both experiments (paired Student’s *t* test, *P* = 0.766; Fig. 4A). As we observed in Robust Aitchison distance comparisons (Fig. 3F), most ddZOTUs were changed at only one timepoint in one cultivar (Fig. S7; Table S2). Furthermore, the interactions between defoliation and sampling timepoint (ANCOM-BC2, fold change >1.5, *P* < 0.01) significantly affected the abundances of 185–715 ZOTUs (Fig. 4A). We also found that sampling time significantly affected the abundance of 294–1,990 ZOTUs (Fig. 4A), which was 3.6–18.9 times more than the number of ddZOTUs, indicating the spatial and temporal dynamics of rice microbiome compositions under field conditions.

We asked which ddZOTUs and bacterial lineages were consistently changed during rice regrowth after defoliation. As shown in Fig. 4B, most ddZOTUs were *Pseudomonadota* (formerly known as *Proteobacteria*), *Myxococcota*, *Bacteroidota*, and *Bacillota* (formerly known as *Firmicutes*), which were also four dominant bacterial phyla in the rhizosphere and root microbiota (Fig. 3E). The top five bacterial orders containing defoliation-enriched ddZOTUs were *Burkholderiales, Methylococcales, Rhizobiales, Myxococcales,* and *Bacteroidales*. The top five bacterial orders containing defoliation-depleted ddZOTUs were *Burkholderiales, Geobacterales, Clostridiales, Pedosphaerales,* and *Bacteroidales* (Fig. 4B). Among all ddZOTUs, only ZOTU849 (*Candidatus Accumulibacter*, family *Rhodocyclaceae*) was consistently enriched in the MH63 communities in the two experiments (Fig. 4C; Table 2; Fig. S8). By contrast, all five depleted ddZOTUs in the NIP communities belonged to *Geobacteraceae* (Table 2). Despite the vast variations in soil bacterial communities in the two experiments, we speculated that the two rice cultivars enriched distinct bacterial taxa for regrowth after defoliation.

**TABLE 2:** Consistently changed ddZOTUs in two experiments

ZOTU ID (family/genus)^*a*^	LFC (MH63)^*b*^		LFC (NIP)^*b*^	
	Exp 1	Exp 2	Exp 1	Exp 2
Root				
ZOTU849 *Candidatus Accumulibacter*	1.66**	1.61**	NA^*c*^	NA
ZOTU129 *Geobacteraceae*	−0.05	−0.69**	−1.11**	−0.7***
ZOTU182 *Geobacteraceae*	−0.61	−0.78***	−1.17**	−0.84***
ZOTU51 *Geobacteraceae*	0.2	−0.33	−0.95***	−0.64***
ZOTU682 *Geobacteraceae*	0.17	−0.46	−1.2***	−0.67**
Rhizosphere				
ZOTU849 *Candidatus Accumulibacter*	1.35**	1.1***	NA	NA
ZOTU182 *Geobacteraceae*	−0.43	−0.24	−0.65***	−0.59**
ZOTU1824 *Clostridium sensu stricto 1*	−0.97**	−1.29***	0.22	−0.71
ZOTU297 *Geobacteraceae*	−0.4	−0.26	−0.66**	−0.59**
ZOTU336 *Clostridium sensu stricto 1*	−0.65**	−0.73**	0.12	−0.89

^*a*^ The taxonomies of ZOTUs are annotated to the family or genus level using 16S rRNA gene sequences.

^*b*^ LFC, log2 transformed fold change (defoliated vs intact) in the designated groups. ** indicates *P* < 0.01, *** indicates *P* < 0.001, ANCOM-BC2, *n* = 12.

^*c*^ NA, data are not available due to low detection rate (detected in <10% samples).

We next investigated the functional compositions of the bacterial communities after defoliation. The bacterial functions were predicted using ZOTU sequences and PICRUST2 (52, 53). A total of 155 KEGG pathways under the “metabolism” and “environmental information processing” catalogs were predicted using ZOTU sequences, and 83 of them were associated with defoliation (Fig. 4C; Table S3; LinDA, *P* < 0.01). Although most pathways showed changes in only one experiment, 7 and 16 KEGG pathways were consistently depleted in the MH63 rhizosphere and root communities, respectively (Fig. 4C and D). Four pathways (Limonene and pinene degradation, Naphthalene degradation, Aminobenzoate degradation, and Caprolactam degradation), which belong to xenobiotics biodegradation and metabolism were depleted in both rhizosphere and root communities. Interestingly, these 19 defoliation-responsive pathways were depleted at 3–4 days after defoliation and were specific for MH63 (Fig. 4D). Taken together, the bacterial functional analysis further indicated that these two rice cultivars enriched or depleted bacteria with distinct functions during regrowth after defoliation.

### Defoliation perturbed specific modules of bacterial co-occurrence networks

**Fig 5:**
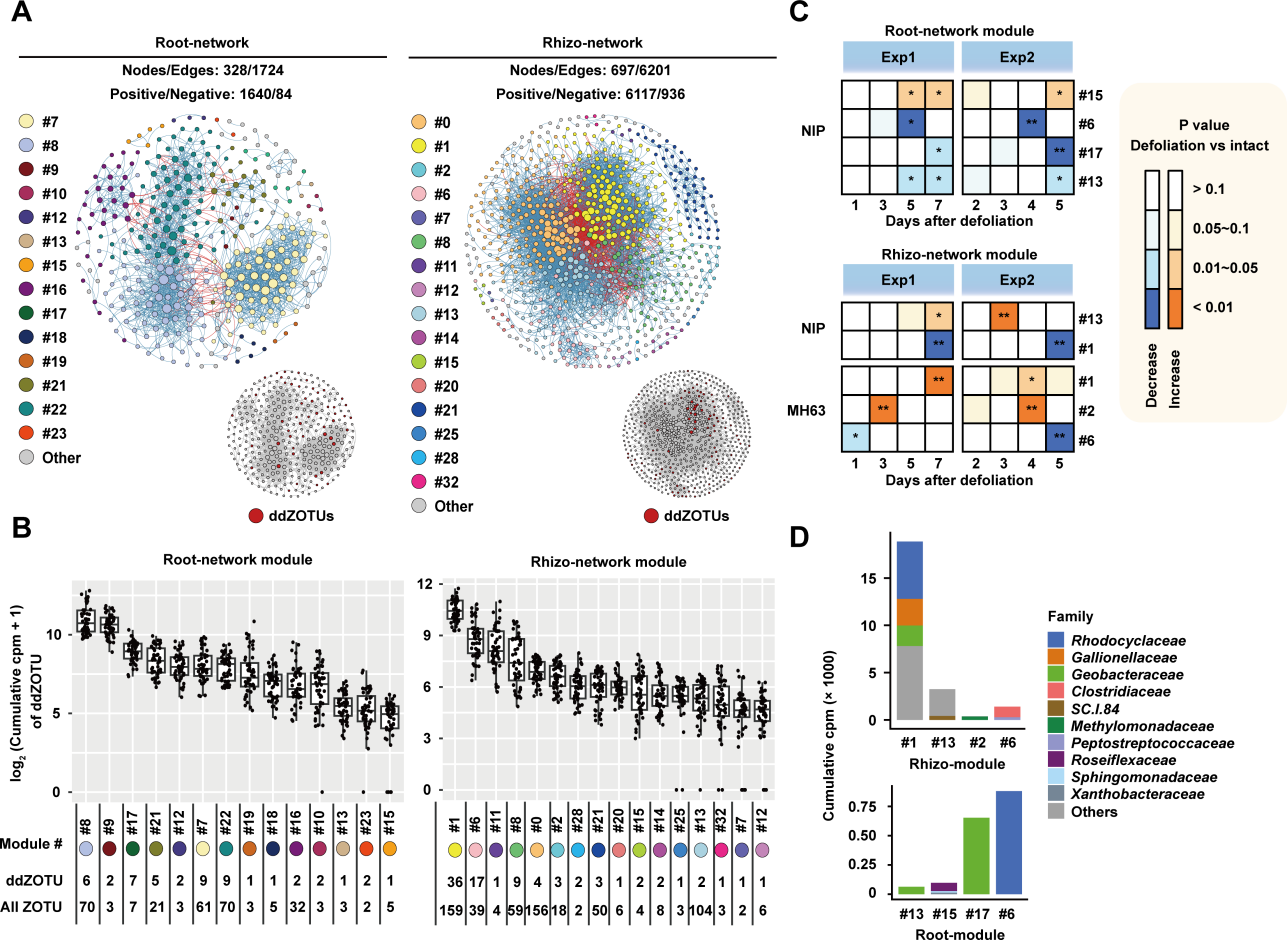
Co-occurrence network modules associated with defoliation treatment. (**A**) Co-occurrence networks of root and rhizo-microbiota. The modules in the networks are differentially colored. The small plots at the bottom right indicate the distribution of ddZOTUs (red points) in the root and rhizo-networks. See also Supporting Information Tables S4 to S6. (**B**) Accumulative abundances of ddZOTUs in each module. Each point in the plot represents a sample. Module ID (#) is shown below the x-axis and labeled with the same color as in Fig. 5A. The numbers of ddZOTUs and ZOTUs (all ZOTUs) in each module are shown below the module ID. (**C**) Heatmaps show the modules that consistently changed after defoliation in the two experiments. * and ** indicate *P* < 0.05 and 0.01, respectively. (**D**) Bar plots showing the mean cumulative cpm value of each module that consistently responded to defoliation. The color of the bars indicates the bacterial families.

The microbial co-occurrence network is a critical feature of the microbial community (54), which provides useful information about microbial interactions (55). To characterize the effects of defoliation on bacterial interactions, we constructed bacterial co-occurrence networks using the significant correlations between ZOTU pairs. We first compared the co-occurrence networks between intact and defoliated plant communities in two compartments. Compared to those of intact plants, the bacterial co-occurrence networks of defoliated plants increased the network topological complexities in terms of edge number and degree in MH63, while the opposite pattern was observed in the NIP networks (Fig. S9). To identify the bacterial subnetworks affected by defoliations, we constructed root and rhizosphere bacterial co-occurrence networks by combining all 16S rRNA gene amplicon data (Fig. 5A, referred to as the root network and rhizo-network). The root network contained 328 nodes (ZOTUs) and 1,724 edges, while the rhizo-network contained 697 nodes and 6,201 edges (Tables S4 and S5), suggesting that rhizosphere bacteria have more complex interactions than root bacteria. Based on network connectivity, the root and rhizo-networks were further divided into 25 and 36 modules (Fig. 5A), respectively, and these modules included bacteria belonging to different phyla (Fig. S10). In the root network, 50 ddZOTUs were found in 14 modules, while in the rhizo-network, 86 ddZOTUs were found in 16 modules (Fig. 5B). Interestingly, most of these ddZOTUs (42%; 36/86) were found in rhizo-module #1, implying that bacterial interactions in this module may be closely associated with rice defoliation. After comparing the z-score-transformed cumulative abundances of the ZOTUs in each module, 69% (42/61) of the co-occurrence modules were affected by defoliation in at least one experiment (*P* < 0.05; Table S6). Among them, eight modules displayed consistent changes in the two experiments (Fig. 5C). The prevalent bacterial taxa in these eight modules belong to 10 families, namely, *Rhodocyclaceae*, *Gallionellaceae*, *Geobacteraceae*, *Clostridiaceae*, *SC.I.84*, *Methylomonadaceae*, *Peptostreptococcaceae*, *Roseiflexaceae*, *Sphingomonadaceae,* and *Xanthobacteraceae* (Fig. 5D). We noticed that the rhizo-module #1 was depleted in NIP communities but enriched in MH63 communities, implying that bacterial taxa in this module likely interacted with defoliated rice in a cultivar-specific manner.

### A bacterial isolate of *Rhodocyclaceae* specifically promoted MH63 regrowth after defoliation

**Fig 6:**
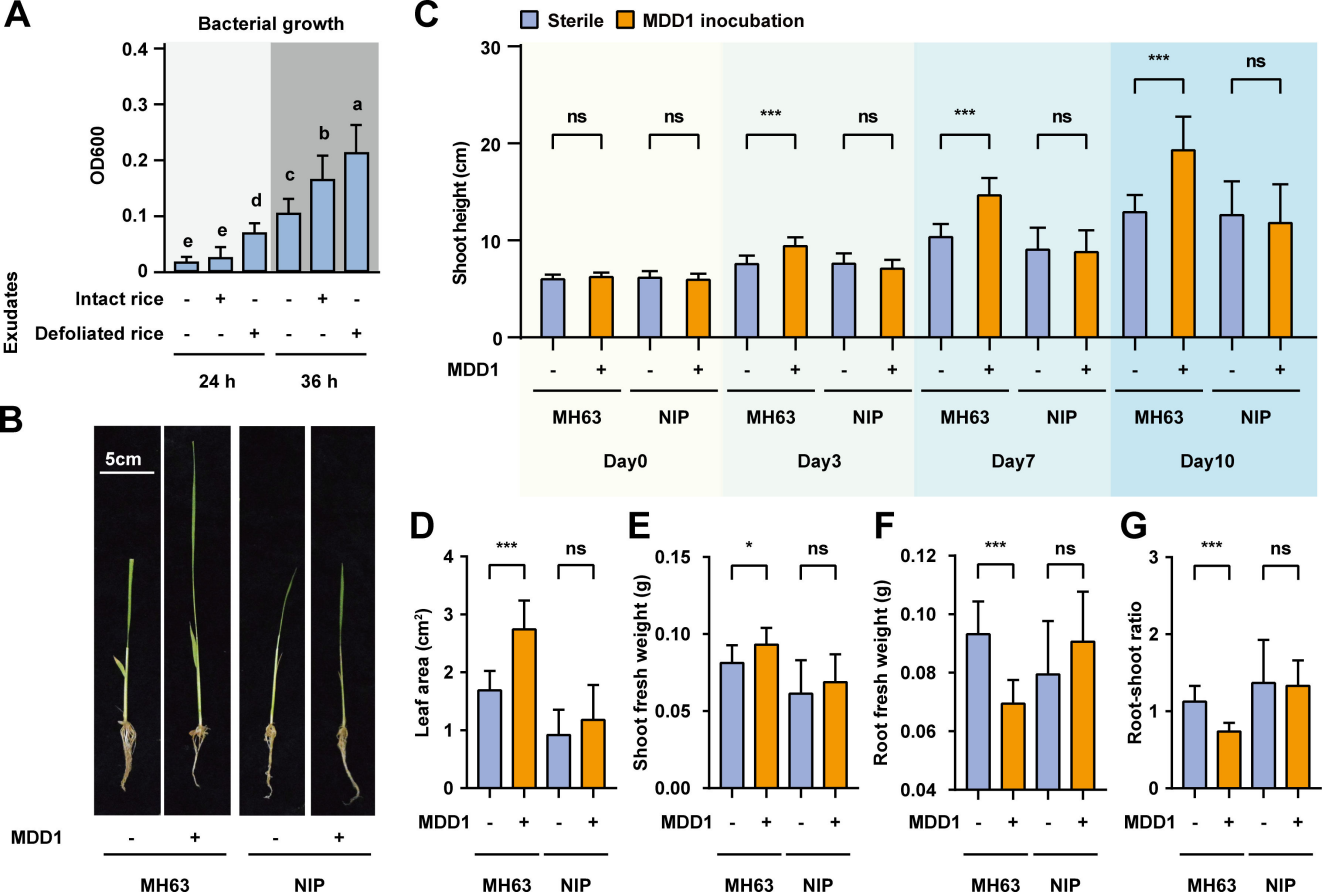
*Rhodocyclaceae* isolate MDD1 displayed variety-specific regrowth activity on defoliated rice plants. (**A**) Effect of root exudates of intact and defoliated rice seedings on MDD1 growth. (**B**) Photograph of defoliated rice seedlings at 10 days after defoliation treatment. (**C**) Comparison of shoot height of defoliated rice seedlings growing with (+) and without (−) MDD1. The data were collected at 0, 3, 7, and 10 days after defoliation. (**D–F**) Comparisons of the leaf area, shoot fresh weight, root fresh weight, and root–shoot ratio of defoliated rice seedlings at 10 days after defoliation. Different letters denote significantly different groups (one-way ANOVA with post hoc Fisher’s Least Significance Difference test, *P* < 0.05, *n* = 8). Error bar, standard deviation; ns, no significant difference; * and *** indicate *P* < 0.05 and 0.001, respectively (Student’s *t* test, *n* = 8).

To verify the function of MH63-enriched bacterial taxa, we examined the regrowth-promoting effects of *Rhodocyclaceae* bacteria since this family was specifically increased in defoliated MH63 roots (Fig. 6; Table 2; Fig. S8) and was also the dominant family presented in Rhizo-module #1 (Fig. 5). Among approximately 1,000 heterogeneous bacterial isolates in high-throughput cultivation (56), we obtained two *Rhodocyclaceae* strains with identical 16S rRNA gene sequences (named MDD1) from MH63 rice roots. According to the 16S rRNA gene sequence, MDD1 belongs to the family *Rhodocyclaceae* species *U. gangwonense* (Fig. S11 and S12). The growth of MDD1 was also stimulated by rice root exudates collected after defoliation (Fig. 6A). We inoculated MDD1 into axenic rice seedlings and then performed the defoliation treatment. Compared with sterile growing plants, inoculation with MDD1 did not increase the shoot heights of MH63 and NIP plants under normal growth conditions (Fig. S13), suggesting that this strain has no growth-promoting effect on intact rice plants. Interestingly, the shoot heights of MDD1-inoculated MH63 plants were 24%, 40.8%, and 47.4% higher than those of sterile stubbles at 3, 7, and 10 days post-defoliation, respectively (Student’s t test, *P* < 0.001; Fig. 6C), indicating that MDD1 promotes the regrowth of MH63 shoots (Fig. 6B and C). At 10 days post-defoliation, the leaf area and shoot weight of MDD1-treated MH63 stubbles were on average 61% and 14.3% higher than those of sterile stubbles, respectively (Fig. 6D and E). By contrast, the fresh weight of MDD1-treated MH63 roots significantly decreased by 25.3% (Student’s t test, *P* < 0.001; Fig. 6F) at 10 days after defoliation. As a result, the root–shoot ratio of MDD1-treated MH63 stubbles was 0.76 ± 0.03 (mean ± SD), which was significantly lower than that of sterile MH63 stubble (1.15 ± 0.06; Student’s t test, *P* < 0.001; Fig. 6G), implying that MDD1 treatment likely reallocates biomass from roots to shoots/leaves to enhance aboveground regrowth. Of note, such regrowth-promoting effects of MDD1 were not observed in defoliated NIP seedlings (Fig. 6B through G ), confirming that the MDD1-mediated enhancement of the regrowth of defoliated rice is dependent on cultivar variety.

## DISCUSSION

Through gnotobiotic growth assays and 16S rRNA gene amplicon sequencing, this study reveals the variety-specific associations between the belowground bacterial communities and the aboveground regrowth ability of defoliated rice plants. We found that defoliation treatment promoted root exudation of MH63 but did not affect that of NIP at the juvenile stage. Under gnotobiotic conditions, we observed mutual growth promotion of MH63 seedlings and the soil bacterium B157 after defoliation treatment (Fig. 1 and 2). Under field conditions, defoliation transiently perturbed the assemblages of bacterial communities of the two rice cultivars at the tillering stage (Fig. 3F), consistent with the fact that defoliated rice plants rapidly recovered photosynthesis capacity as new leaves regrew from stubbles (Fig. S3). The distinct changes in the microbiota and the different regrowth abilities of defoliated NIP and MH63 suggest that host genetics is the dominant factor in determining the assemblages of beneficial microbiota upon defoliation. According to the ddZOTUs and co-occurrence network modules that were specifically enriched in defoliated MH63 plants, we hypothesized that *Rhodocylaceae* was the key taxon for rice regrowth after defoliation. Indeed, MDD1, which belongs to the genus *Uliginosibacterium* in the family *Rhodocyclaceae*, specifically promoted the regrowth of defoliated MH63 but had no effect on NIP plants (Fig. 6). These findings highlight that belowground microbiota play critical roles in plant recovery from foliar damage and provide novel insights into the recruitment of rhizosphere bacteria upon mechanical defoliation.

The role of microbiota in plant disease resistance and stress tolerance has been widely explored, and the cry-for-help hypothesis was proposed to explain the active selection of rhizospheric and endospheric bacteria to defend against pathogens (18, 25). Under biotic stresses, disease-resistant cultivars recruit soil bacteria to suppress pathogens and benefit subsequent plant generation (19, 20, 57, 58). Under abiotic stress, for example, drought shifts the microbial community in the rhizosphere and triggers the enrichment of beneficial bacterial taxa to increase the drought tolerance of plants (15, 59). In addition to plant disease resistance and abiotic stress tolerance, our results illuminate that the rhizosphere and root microbiome are crucial for plant regrowth after foliar injuries such as defoliation. We observed that rice seedlings indeed regrew faster in germy soil than in sterile soils (Fig. 2). This finding is consistent with a recent study in *Arabidopsis* in which root microbiota rescued *Arabidopsis* growth under low photosynthetically active radiation (60). Interestingly, defoliation-triggered enrichment and depletion of rhizosphere and root bacteria were distinct between NIP and MH63 (Fig. 4 and 5). Despite the spatial and temporal dynamics of the rice microbiome under field conditions (Fig. 3D; Table 2), we observed that 16 and 7 bacterial pathways were constantly depleted by defoliation in MH63 but not in NIP (Fig. 4), implying that the two cultivars have different abilities to recruit a beneficial microbiome upon defoliation. In addition, MH63 and NIP had significantly different root exudates (Fig. 1C) and regrowth vigor (Fig. 3C) after defoliation. The high regrowth ability of MH63 was also observed in ratooning rice (61). These data suggest that MH63 resembles a defoliation-tolerant variety. Consistent with the cry-for-help hypothesis from microbiome-plant‒pathogen interactions, the defoliation-tolerant variant can recruit a regrowth-promoting bacterial microbiota upon defoliation.

Intriguingly, the *Uliginosibacterium* strain MDD1 specifically promoted MH63 regrowth after defoliation but had no effect on defoliated NIP seedlings or intact rice plants (Fig. 6). Previously, a cultivar-dependent response to bacterial seed treatment under gnotobiotic conditions was reported in *Brassica napus* (62). L. Xu et al. (59) observed associations between monoderm bacteria and drought in sorghum. To the best of our knowledge, the beneficial associations between a bacterial genus and a crop cultivar, as we found in this study, have not been reported before. The gnotobiotic growth assays revealed that MDD1 promoted leaf growth while reducing the root weight of defoliated MH63 seedlings (Fig. 6F and G). This finding is consistent with the observation that defoliation enhanced root exudation in MH63 plants (Fig. 1C). We speculated that defoliation increased MH63 root exudation and thereby enhanced the colonization of beneficial bacteria such as B157 and MDD1. As a reward, MDD1 and other bacteria reinforced the reallocation of biomass from roots to shoots, which prioritized leaf growth over storage in defoliated MH63 plants. Plants also reallocate biomass between sources and sinks to adapt to environmental stresses such as drought (63). It will be interesting to test whether MDD1 promotes rice regrowth after abiotic stresses in the future. Furthermore, the root–shoot ratio is a complex trait affected by environmental stress (64), and a previous genome-wide association study identified 35 loci contributing to the variations in the root–shoot ratio of different rice cultivars (65). The identification of the MH63-specific regrowth-promoting bacterium MDD1 implies that host genetics and rhizospheric microbiota collaboratively regulate rice resilience to defoliation and, potentially, other environmental stresses.

The importance of the microbiome for crop productivity is highly recognized (17, 66); however, there are still many challenges in identifying and verifying the beneficial associations from a tremendous number of soil bacteria and different crop varieties in field trials. In this study, owing to the temporal and spatial heterogeneity of soil physiochemical properties and bacterial communities, considerable variations were presented between microbiome data obtained from the same field in 2 years (Fig. 3D). This is a common phenomenon in soil microbiome studies (7). Despite these findings, we observed that defoliation exerted a consistent effect on the rhizosphere and root bacterial compositions (Table 1). We detected eight ddZOTUs that showed consistent changes in Exp1 and Exp2 (Table 2), while most ddZOTUs were detected in only one year in this study (Fig. S7). This is likely due to the functional redundancy between different bacteria (67). By taking advantage of recent progress in bacterial function prediction using 16S rRNA gene amplicon-based sequencing data (68), the enriched bacterial functions provide a biologically relevant interpretation for our 2-year microbiome data (Fig. 4D). The co-occurrence network analysis further indicated that defoliation treatment enhanced bacteria–bacteria interactions in MH63 (Fig. S9) and identified three bacterial co-occurrence modules in response to defoliation (Fig. 5). According to 16S rRNA gene metagenomics, *Rhodocyclaceae* and three other bacterial families (Fig. 5D) emerged as key bacterial taxa associated with rice regrowth after defoliation. Indeed, the *Rhodocyclaceae* strain MDD1 from MH63 roots displayed variety-specific regrowth-promoting activity (Fig. 6), demonstrating the strengths of bacterial metagenomics for identifying key bacterial taxa from field trials. However, in our attempts at root bacteria isolation, we currently do not obtain other strains belonging to defoliation-related bacteria except MDD1. Therefore, the function of most ddZOTUs in rhizo-module #1 has not yet been tested. Application of culturomic technology, such as targeted isolation and cultivation of bacterial taxa of interest (69), is highly demanded to isolate those defoliation-associated bacteria. Nevertheless, MDD1 provides an opportunity to investigate defoliation-triggered interactions between soil bacteria and rice varieties. In the future, the integration of exometabolomics (70, 71), synthetic bacterial communities (72, 73), and plant genetics is necessary to dissect the communications between MDD1 and defoliated rice plants.

### Conclusion

In summary, this study found that belowground microbiota play a crucial role in rice regrowth after defoliation. We show here that two cultivated rice varieties have different root exudates and enriched distinct bacterial taxa after defoliation. According to these 16S rRNA gene metagenomics data, we obtained the *Uliginosibacterium* strain MDD1, which specifically promoted MH63 regrowth after defoliation. Such variety-specific associations between a bacterial strain and a rice cultivar provide novel insight into microbiome‒root‒shoot communications. The findings presented in this study will help to engineer the microbiome to improve rice regrowth after defoliation and environmental stresses.

## MATERIALS AND METHODS

### Root exudate collection and TOC measurements

Rice seeds were sterilized in 75% ethanol for 30 s followed by 0.1% HgCl_2_ for 10 min. After rinsing with sterilized distilled H_2_O five times, the surface-sterilized rice seeds were placed on a sterilized wet paper tower in Petri dishes and germinated in a growth chamber for 5 days at 28°C. Germinated 5-day-old sterile rice seeds were transferred to a sterilized hydroponic culture solution containing 1.43 mM NH_4_NO_3_, 0.51 mM K_2_SO_4_, 1.64 mM MgSO_4_, 0.32 mM NaH_2_PO_4_, 1 mM CaCl_2_, 0.17 mM NaSiO_3_, 0.27 µM CaSO_4_, 18.88 µM H_3_BO_3_, 9.47 µM MnCl_2_, 0.15 µM ZnSO_4_, 70.79 mM C_6_H_8_O_7_, 29.94 nM (NH4)_6_Mo_7_O_24_, 0.02 mM EDTA, and 16.65 µM FeSO_4_. The rice seedlings were grown in a growth chamber for 14 days with a day/night cycle of light/28°C for 16 h and dark/25°C for 8 h. Then, the rice seedlings were rinsed thoroughly with distilled water and transferred to 15 mL tubes filled with 8 mL of H_2_O to collect the root exudates. After 18 hours, the exudate solutions were filtered through a 0.22-µm membrane and subjected to TOC analysis using a TOC-L CPH/CPN (Shimadzu Corporation, Japan).

### Measurement of bacterial growth

To assess the effect of defoliation on rhizosphere bacterial growth, sterilized rice seedlings were prepared as described above. The rice seedlings were grown in 1/2-strength Murashige and Skoog (MS) media (Phytotech Labs, Lenexa, USA) in a growth chamber. Of note, sucrose was not added to the MS medium in cocultivation assays in this study. After 2 weeks, rice shoots were defoliated by cutting the shoots at a height of 6 cm. The *Serratia marcescens* strain B157 was grown in 1/10 tryptic soy broth (Solarbio, China) media at 28°C. The overnight culture of B157 was collected and washed twice with H_2_O. The bacterial cells were resuspended in H_2_O with an optical density at 600 nm (OD_600_) of 0.2, which approximately equals 6 × 10^8^ cell/mL. For co-cultivation, the bacterial susceptions were inoculated into liquid MS media at a 1:100 dilution. After 6 days of cocultivation, the OD_600_ of the media was measured using a spectrophotometer. The experiments were repeated twice with at least eight replicates each time.

### Measurement of rice regrowth after defoliation

To measure the regrowth of the rice plants after defoliation, the rice seeds were sterilized and germinated for 7 d as described above. Seven-day-old sterile rice seedlings were placed in a tissue culture bottle containing 8 g of sterilized soil and 8 mL of sterilized distilled water. To prepare the soil slurry, 2 g of paddy soil was mixed with 10 mL of distilled water. Then, 1 mL of soil slurry was added to each bottle. For the control groups, the same amount of filter-sterilized soil slurry was added to treat the rice plants. After 1 week, rice seedlings were defoliated by cutting the shoots in a germ-free hood. The shoot height and leaf area of rice seedlings were measured 1 week after defoliation.

*U. gangwonense* MDD1 was isolated from MH63 roots using a high-throughput culturation method (56). To test the effect of the MDD1 strain, 5-day-old sterilized rice seedlings were prepared in MS media as described above. The MDD1 isolate was subsequently grown in 1/10 tryptic soy broth (Solarbio, China) media at 28°C. MDD1 bacterial cells were collected from overnight culture by centrifugation and washed twice with distilled water. Then, the bacterial cells were resuspended in H_2_O, and the cell density was adjusted to OD_600_ = 0.5 (approximately 8 × 10^6^ cell/mL). For MDD1 inoculation, rice roots were immersed in the bacterial suspensions for 1 h and then transferred to sterile soil. Defoliation treatment was performed at 10 days post-inoculation by cutting the shoot at 7 cm above the ground. The regrowth of the rice plants was assessed by measuring the length and weight of the shoots and roots at 3, 7, and 10 days after defoliation.

To measure the effect of defoliation on bacterial growth in the field (Fig. 1F), 2-month-old rice plants were subjected to defoliation treatments, and root samples were collected 3 days after defoliation. The root samples were ground in 10 mM sterilized MgCl_2_ solution, and suspensions were diluted and plated on 1/10 tryptic soy agar plates. The number of colonies was counted after incubation at 28°C for 3 days.

### Defoliation treatment of rice plants in paddy fields

For field experiments, MH63 and NIP plants were grown in the field at the campus of Huazhong Agricultural University, Wuhan, China (30°28′27″ N, 114°20′57″ E). Rice seeds were dehulled and surface sterilized in 75% ethanol for 30 s and then in 0.1% HgCl_2_ for 10 min. After being rinsed five times with sterilized water, surface-sterilized seeds were germinated and grown on MS agar plates for 15 days. Then, rice seedlings were transferred to the paddy field. For defoliation treatment, the leaves of 6-week-old plants were cut using scissors at 25 cm above the ground. In this study, roots with mud at 5–10 cm below ground were collected with three replicates. The experiments were performed in the same field for two consecutive years (2018 and 2019, referred to as Exp1 and Exp2, respectively).

### DNA extraction and amplicon sequencing

To collect rhizospheric and endospheric fractions, we followed the protocol for *Arabidopsis* microbiome analysis (74) with modifications (75). After removing loosely attached soil and briefly rinsing with distilled water, the root materials were vortexed in phosphate-buffered saline with 0.1% Tween 20 (PBST). Then, the soil particles in PBST were collected by centrifugation and used as rhizosphere fractions. The remaining root samples were further thoroughly washed three times with PBST and used as root fractions.

For bacterial DNA extraction, the samples were ground in liquid nitrogen and then extracted using a DNeasy PowerSoil Pro Kit (QIAGEN, Germany) following the manufacturer’s instructions. The 16S rRNA gene amplicon libraries were prepared using a modified two-step PCR procedure as we described previously (75). In the first PCR, the V5-V6-V7 region of the 16S rRNA gene was amplified using primers Rd1-f6-799F and Rd2-f6-1193R. Touchdown PCR was performed using the following program: 94°C for 3 min; 34 cycles of 94°C for 1 min, annealing at 60°C–52°C for 1 min, and 72°C for 45 s; and 72°C for 10 min. In this PCR program, the annealing temperature was set to 60°C for four cycles, 58°C for six cycles, 56°C for eight cycles, 54°C for eight cycles, and 52°C for eight cycles. After purifying the PCR products with Agencourt AMPure XP beads (Beckman Coulter Life Sciences, IN, USA), 60 ng of the amplicon was digested using Cas9 endonuclease and gRNA1171 to remove the abundant 16S rRNA gene sequences of plant organelles (75). Then, the second index PCR was performed in a 25 µL reaction containing 20 ng of the Cas9-digested products, 12.5 µL of I-5 2 × High-Fidelity Master Mix (Molecular Cloning Laboratories, CA, USA), and 1.25 µL of P5-index-bc1 (10 µM) and P7-index-bc2 (10 µM). The sequences of P5-index-bc1 and P7-index-bc2 are 5′-AATGATACGGCGACCACCGAGATCTACACXXXXXX TCGTCGGCAGCGTCAG-3′ and 5′-CAAGCAGAAGACGGCATACGAGAT XXXXXX GTCTCGTGGGCTCGGAG-3′ (XXXXX is the 6-mer index sequence), respectively. The PCR program was as follows: 98°C for 1 min; 10 cycles of 98°C for 30 s, 58°C for 30 s, and 72°C for 1 min; and 72°C for 5  min. The PCR products were purified using AMPure XP beads (Beckman Coulter Life Sciences, IN, USA). The pooled amplicons were sequenced with an Illumina HiSeq 2500 using a 2 × 250 bp paired-end run (Novogene, China). During DNA extraction and amplicon library preparations, negative control reactions were always included to ensure no contamination from reagents, primers, and plastic consumables.

### Bioinformatics analysis

The 16S rRNA gene amplicon reads were processed using VSEARCH (48) and UNOISE3 (47). Briefly, paired-end reads of 16S rRNA gene amplicons were merged and quality filtered using VSEARCH (48). After clipping the primer sequence and removing short (<150 bp) and low-abundance reads (count <8), merged sequences were denoised to exact sequence variants as ZOTUs (zero-radius operation taxonomic units) with UNOISE3 (47). The chimeras in ZOTUs were identified and removed using VSEARCH. The taxonomy of each ZOTU was assigned using the q2-feature-classifier plugin in QIIME 2 (76) based on the Silva reference database (SILVA Release 138, Ref NR99, https://www.arb-silva.de/). According to the newly revised nomenclature of bacterial phyla by International Code of Nomenclature of Prokaryotes (46), *Proteobacteria* and *Firmicutes* are renamed as *Pseudomonadota* and *Bacillota*, respectively. The ZOTUs annotated as organelle 16S rRNA gene sequences were removed, and the resulting ZOTU table was used for further analysis.

### Statistical analysis

All statistical analyses and graphics were performed using R software version 4.0.2 (http://www.r-project.org), ImageGP (77), and EasyAmplicon (78). The ZOTU table was rarefied to 15,033 reads per sample for bacterial diversity analysis. The α diversity indices were calculated using the Phyloseq package (version 1.42.0) (79). Permutational multivariate analysis of variance (PERMANOVA) was employed to test the effects of variety, sampling timepoint, and defoliation on bacterial communities using the ADONIS function in the vegan package (version 2.6–4). Nonmetric multidimensional scaling (NMDS) was used for ordination based on the UniFrac phylogenetic distance matrix for bacterial community structure using the Phyloseq package. Differences of relative abundances of ZOTUs were assessed using the “ancom-bc2” function with covariates of sampling timepoints (fold change >1.5, *P* < 0.01). PICRUST2 (52) was used to predict the functions of microbial taxa based on 16S rRNA gene sequences. Depleted or enriched KEGG pathways under “metabolism” and “environmental information processing” were analyzed using the linear models for differential abundance analysis (LinDA) function in ggpricrust2 (53).

### Co-occurrence network analysis

Bacterial co-occurrence network analysis was performed as described in (9, 80). Briefly, the ZOTU abundances were normalized to CPM (counts per million) using EdgeR (81), and then Spearman’s correlation coefficient (*r*) was calculated for all ZOTUs. ZOTU pairs with |*r*| > 0.8 and FDR-adjusted *P* < 0.001 were considered valid co-occurrence correlations. The modules in co-occurrence networks, which are the substructures of nodes with a greater density of edges within groups than between them, were identified using the Louvain algorithm in Gephi (https://gephi.org/). We first compared the co-occurrence networks between intact and defoliated plants using rhizosphere and root bacterial communities separately. Then, two co-occurrence networks covering all rhizosphere and root bacterial communities were constructed. To identify differential modules, the relative abundances of each module were transformed to z scores, and then Welch’s t test was used to determine the significantly enriched or depleted modules after defoliation.

## Data Availability

The 16S rRNA gene sequencing data are deposited at the National Genomics Data Center of China under accession number PRJCA015450. The computer scripts used in this study are deposited in GitHub (https://github.com/JiangChangjin1/Defoliation-root-microbiome-silva138.git).

## SUPPLEMENTAL MATERIAL

Supplemental figures spectrum.01254-24-s0001.docx Fig. S1 to S13.

Supplemental tables spectrum.01254-24-s0002.xlsx Tables S1 to S6.

ASM does not own the copyrights to Supplemental Material that may be linked to, or accessed through, an article. The authors have granted ASM a non-exclusive, world-wide license to publish the Supplemental Material files. Please contact the corresponding author directly for reuse.
