# Supplementary material for: Two rice cultivars recruit different rhizospheric bacteria to promote aboveground regrowth after mechanical defoliation
Source: Microbiol Spectr. 2024 Dec 9;13(1):e01254-24. doi: 10.1128/spectrum.01254-24 (PMC11705949; doi:10.1128/spectrum.01254-24)
Supplement: Supplemental figures — Fig. S1 to S13. [file spectrum.01254-24-s0001.docx]

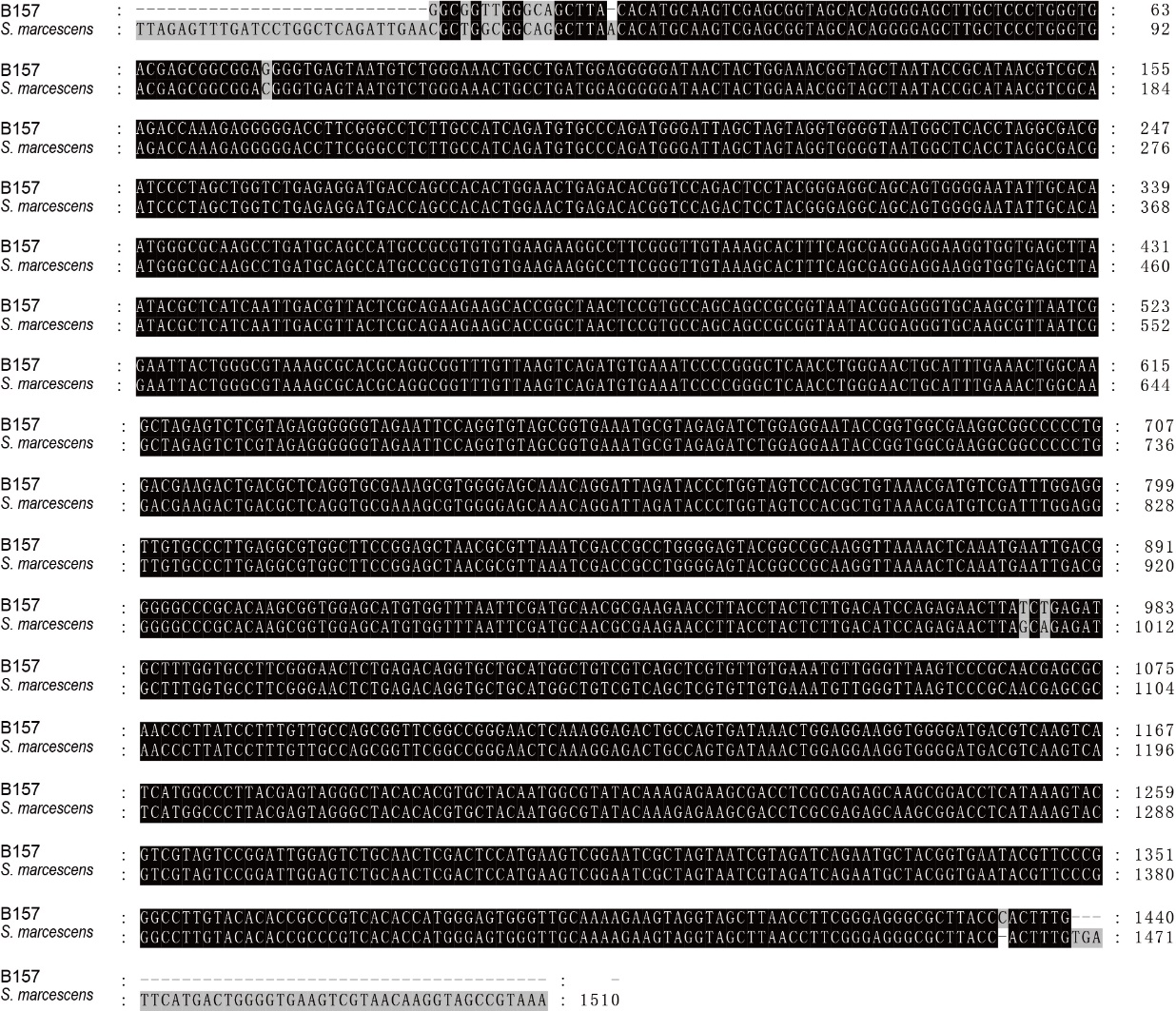


**Figure S1.** Alignment of the 16S rRNA gene of B157 and *S. marcescens* strain S-JS1. The NCBI accession number of 16S rRNA gene of S-JS1 is KJ427750.


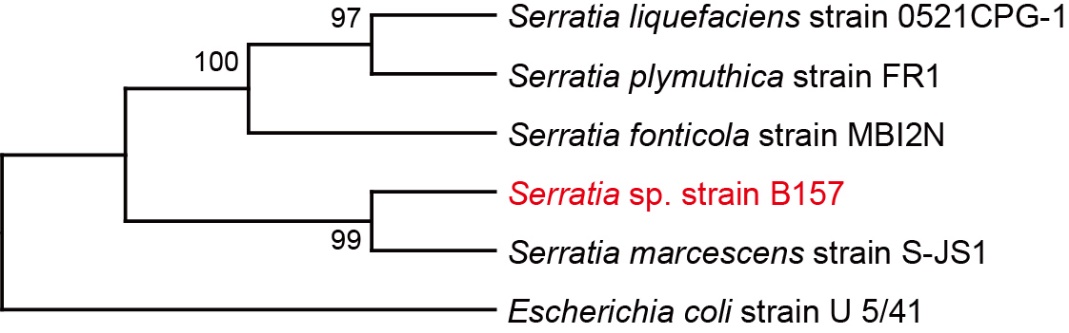


**Figure S2.** Phylogenetic tree of B157 and related *Serratia* strains based on the full-length 16S rRNA gene sequences. The tree was constructed using the Neighbor-joining (NJ) method in MEGA. Numbers at nodes indicate bootstrap support values for 1000 replicates. *E. coli* was used as outgroup. The accession numbers of the 16S rRNA gene sequences used in this phylogenetic analysis are as follows: *Serratia liquefaciens* strain 00521CPG-1, MN107882.1; *Serratia plymuthica* strain FR1, OR083428.1; *Serratia fonticola* strain MBI2N, OR225661.1; *Serratia marcescens* S-JS1, KJ427750.1; *Escherichia coli* strain U 5/41, ON799228.1.


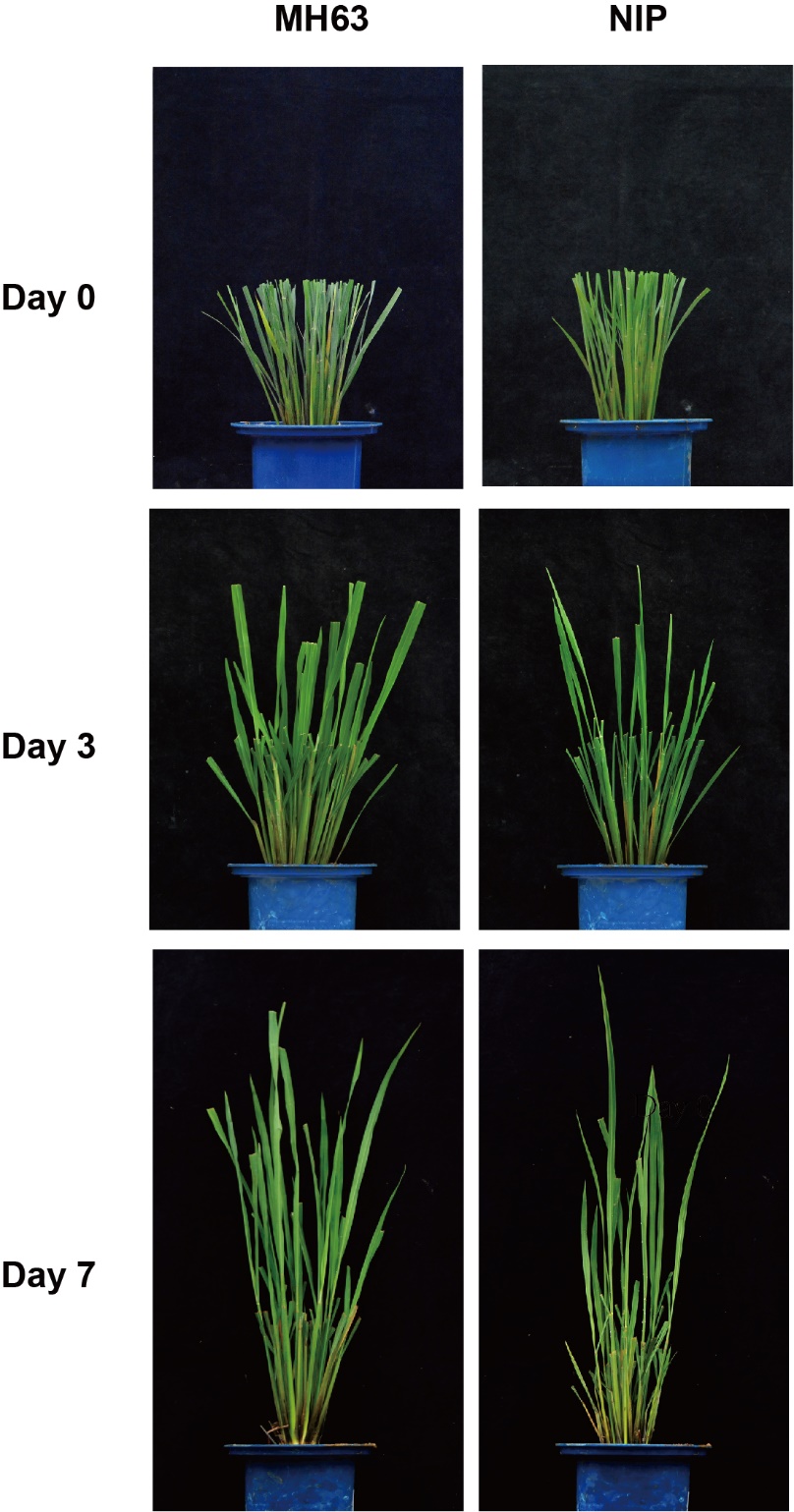


**Figure S3.** Photos showing the regrowth of rice plants after defoliation treatments in paddy fields.

**
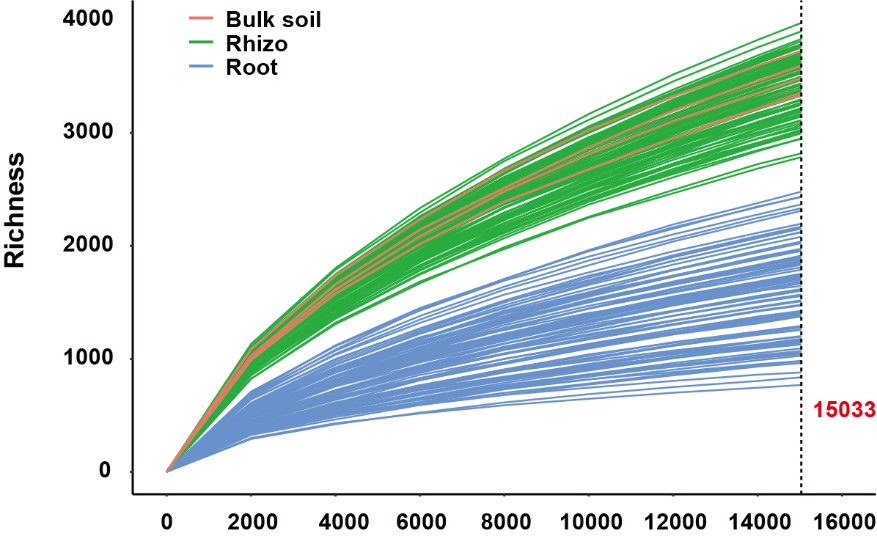
**

**Figure S4.** Rarefaction curves showing bacterial richness and 16S rRNA gene sequencing depth for all samples. The dashed vertical line indicates the sequence number used for normalization of ZOTU table for bacterial diversity analysis.


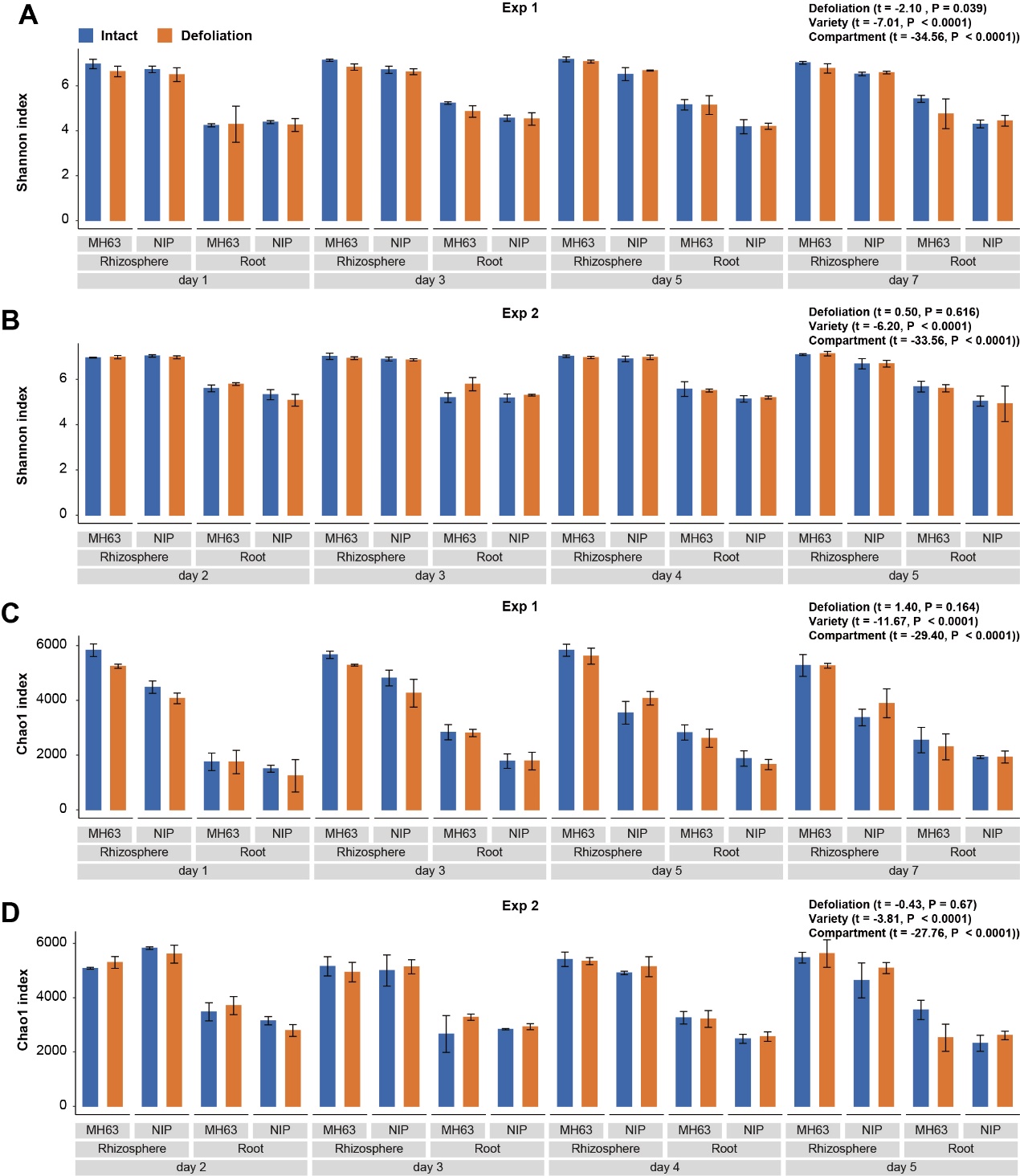


**Figure S5.** Influence of defoliation, variety and compartment on α diversities of bacterial microbiome. Linear mixed effect model was used to evaluate the impact of defoliation, variety and compartment on α diversities of bacterial communities with sample timepoints as the random effect.

**
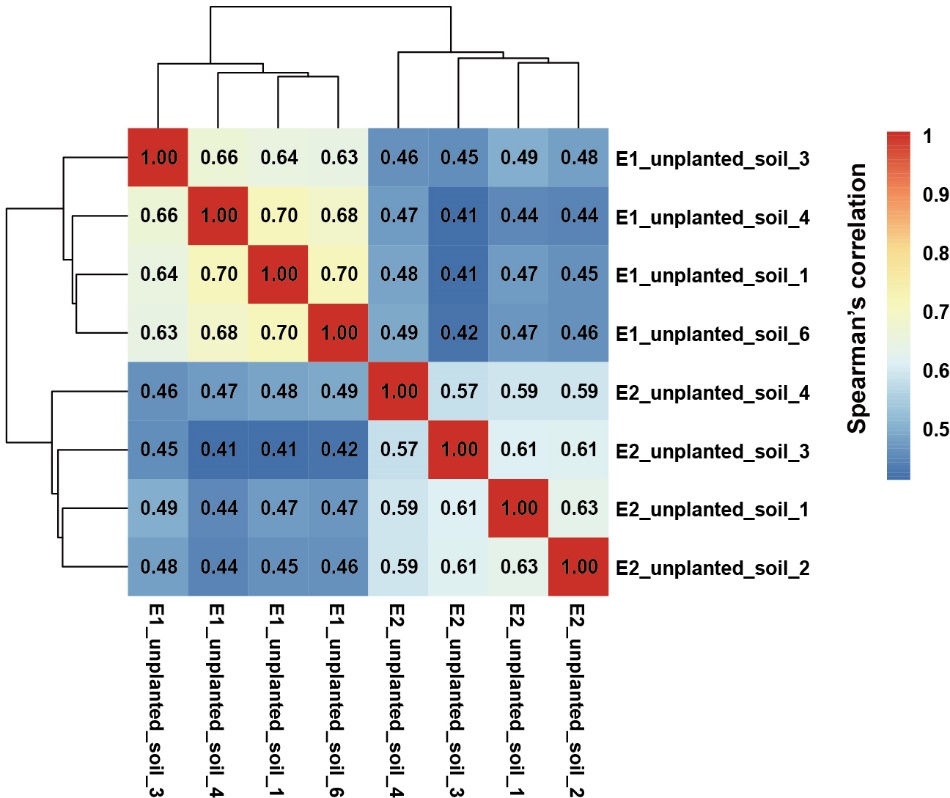
**

**Figure S6.** Correlation coefficients of bulk soil ZOTUs in two experiments. E1 and E2 indicate Exp1 and Exp2.

**
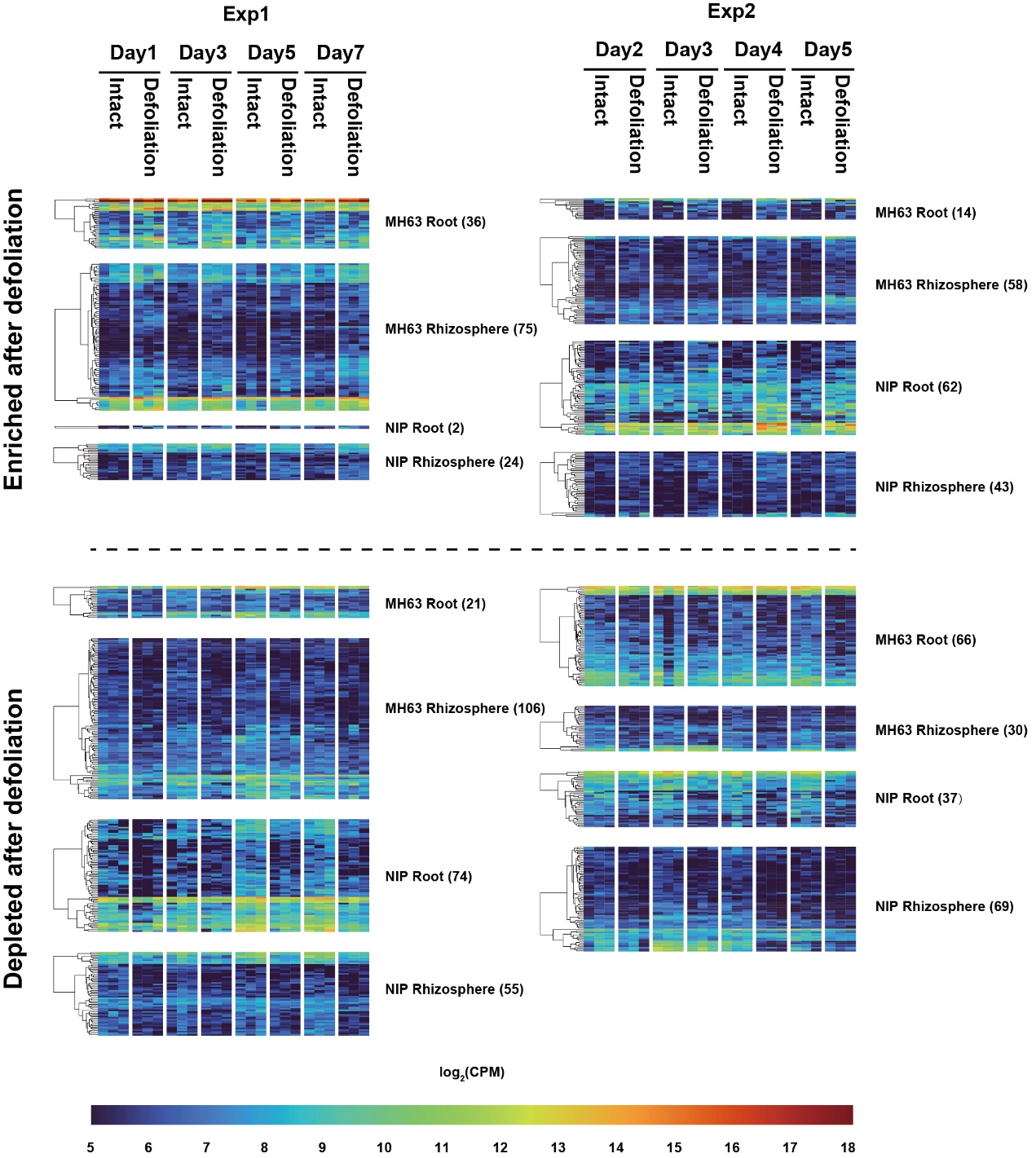
**

**Figure S7.** Heatmap showing differential ZOTUs that significantly changed after defoliation treatment in MH63 and NIP in the two experiments. The number of ddZOTUs is shown in brackets. CPM, count per million reads.


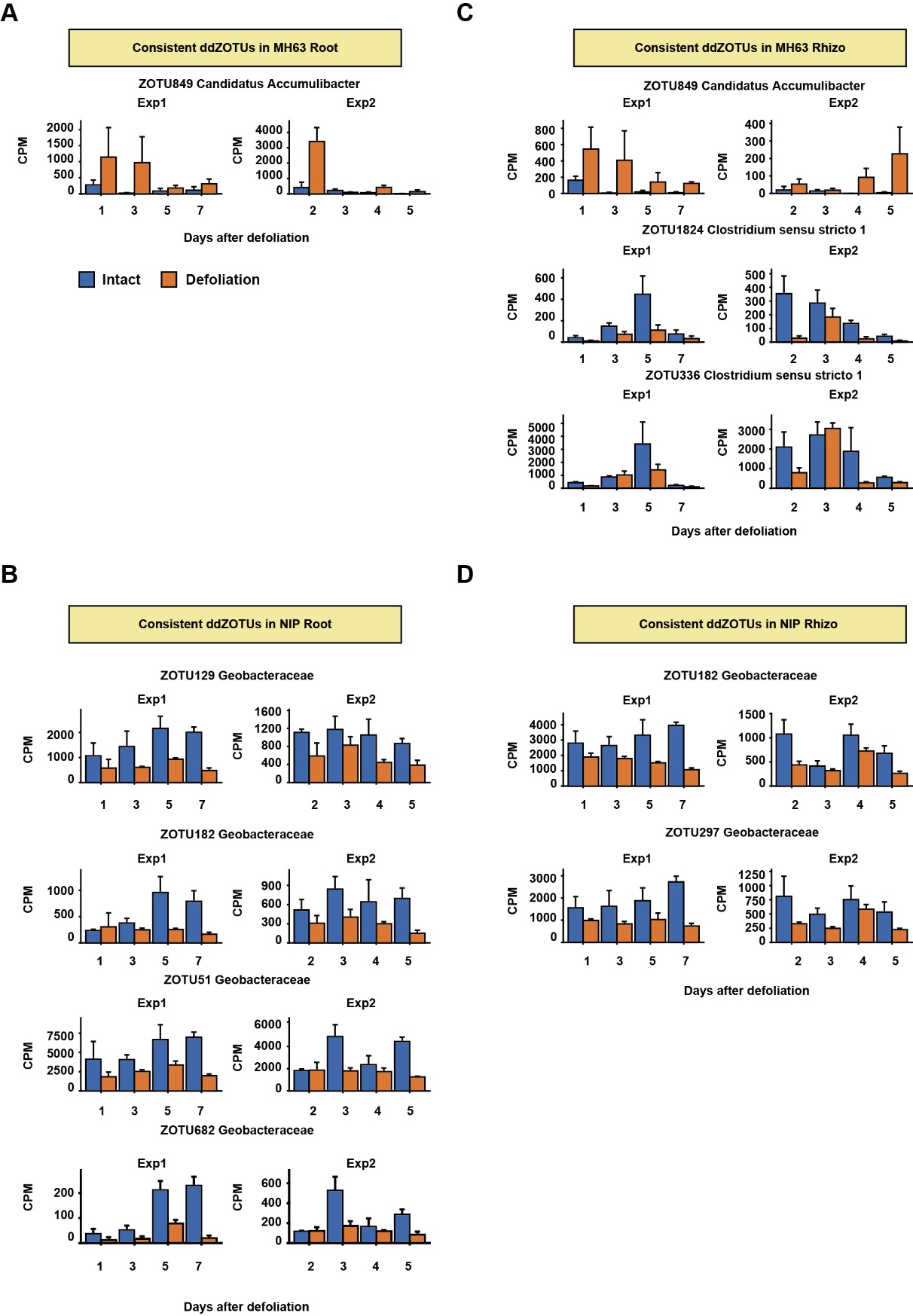


**Figure S8.** Comparisons of the abundances of ddZOTUs that were consistently enriched or depleted in two experiments. The y-axis indicates CPM (count per million) of ZOTU. Error bar, standard deviation. The taxonomic annotations of ZOTU are shown as below (Phylum, Class, Order, Family, Genus):

ZOTU849: *Pseudomonadota* (formerly known as *Proteobacteria*), *Gammaproteobacteria*, *Burkholderiales*, *Rhodocyclaceae*, *Candidatus Accumulibacter*.

ZOTU1824:  *Bacillota* (formerly known as *Firmicutes*), Clostridia, *Clostridiales*, *Clostridiaceae*, *Clostridium sensu stricto 1*.

ZOTU336: *Bacillota* (formerly known as *Firmicutes*), *Clostridia*, *Clostridiales*, Clostridiaceae, *Clostridium sensu stricto 1*.

ZOTU129: *Desulfobacterota*, *Desulfuromonadia*, *Geobacterales*, *Geobacteraceae*, uncultured.

ZOTU182: *Desulfobacterota*, *Desulfuromonadia*, *Geobacterales*, *Geobacteraceae*, uncultured.

ZOTU51: *Desulfobacterota*, *Desulfuromonadia*, *Geobacterales*, *Geobacteraceae*, uncultured.

ZOTU682: *Desulfobacterota*, *Desulfuromonadia*, *Geobacterales*, *Geobacteraceae,* uncultured.

ZOTU297: *Desulfobacterota*, *Desulfuromonadia*, *Geobacterales*, *Geobacteraceae*, uncultured.

**
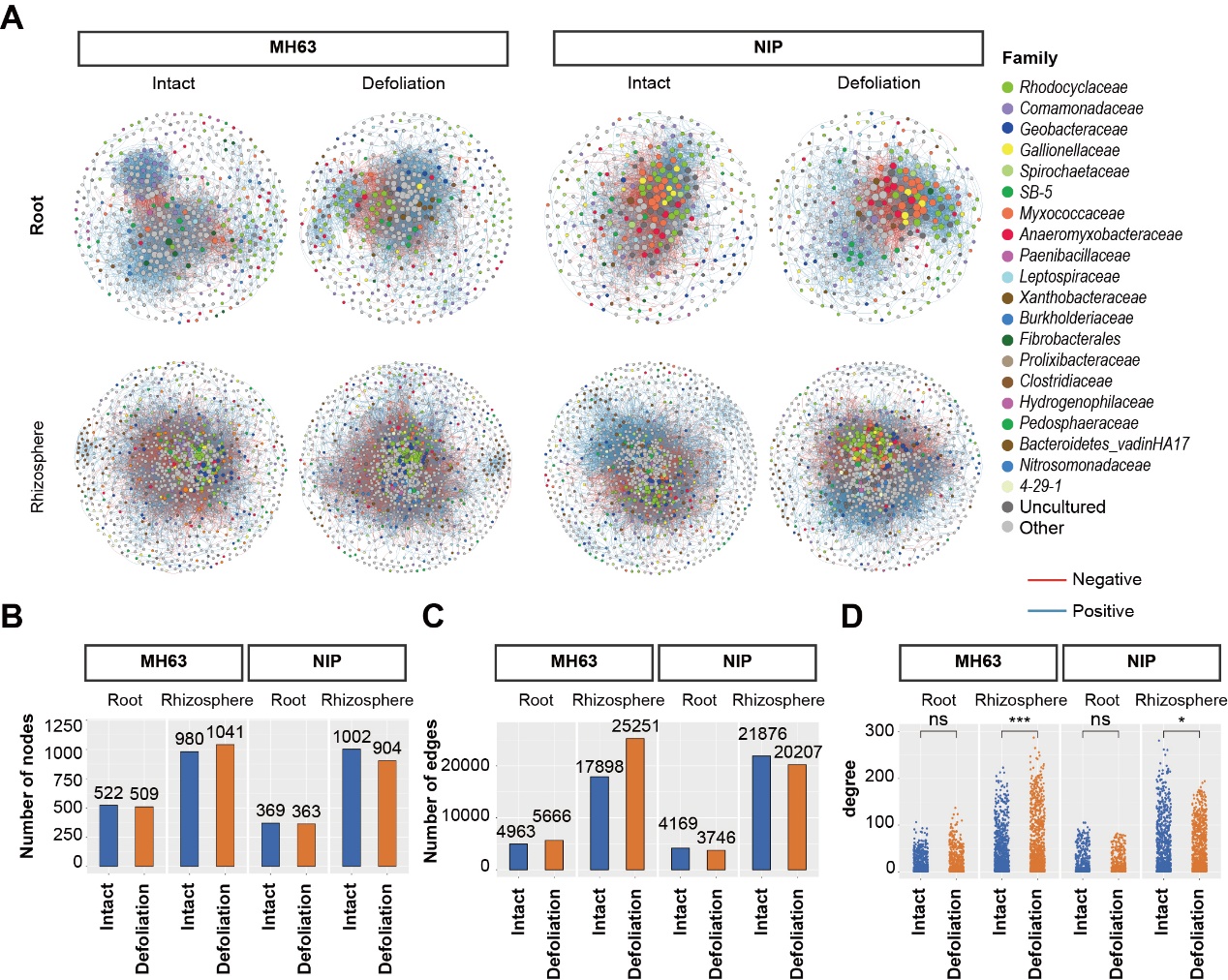
**

**Figure S9.** Comparisons of co-occurrence networks of bacterial communities of intact and defoliated rice plants. (A) Co-occurrence network of intact and defoliated communities of two rice variants. The node (ZOTU) color indicates bacterial families; node size indicates the degree of connection. Edge color represents positive (blue) and negative (red) correlations. (B-D) Comparison of the node number (B), edge number (C) and degree (D) of bacterial networks of intact and defoliated plants. * indicates p < 0.05；*** indicates p < 0.001 (Wilcoxon rank sum test).

**
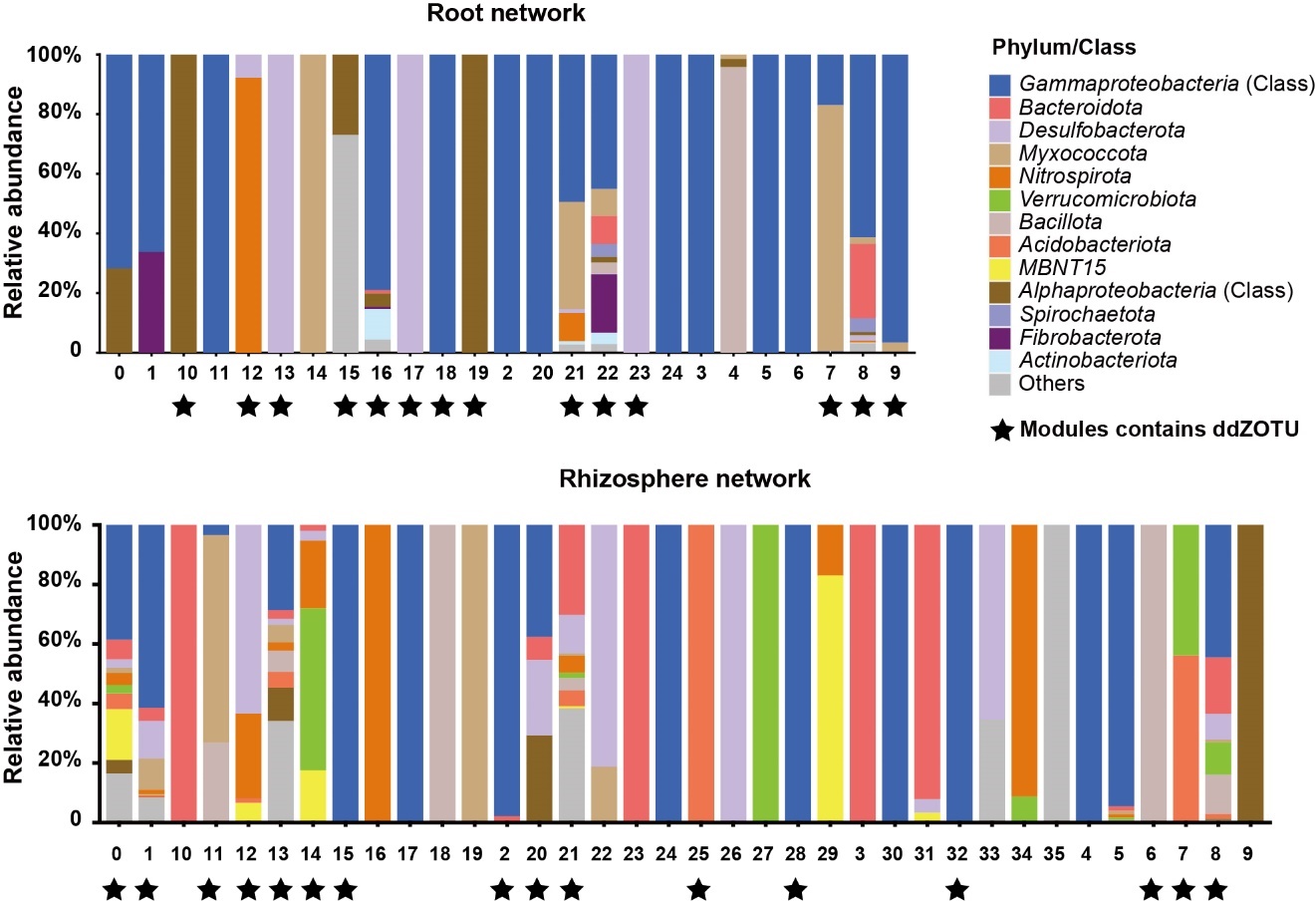
**

**Figure S10.** Bar plot showing the relative abundances of bacterial phyla in different modules of root- and rhizo-networks. The stars represent the network modules containing ddZOTUs. *Pseudomonadota* (formerly known as *Proteobacteria*), including *Alphaproteobacteria* and *Gammaproteobacteria*, are shown at class level. *Bacillota* (formerly known as *Firmicutes*) are new phyla names from Oren & Garrity (2021).


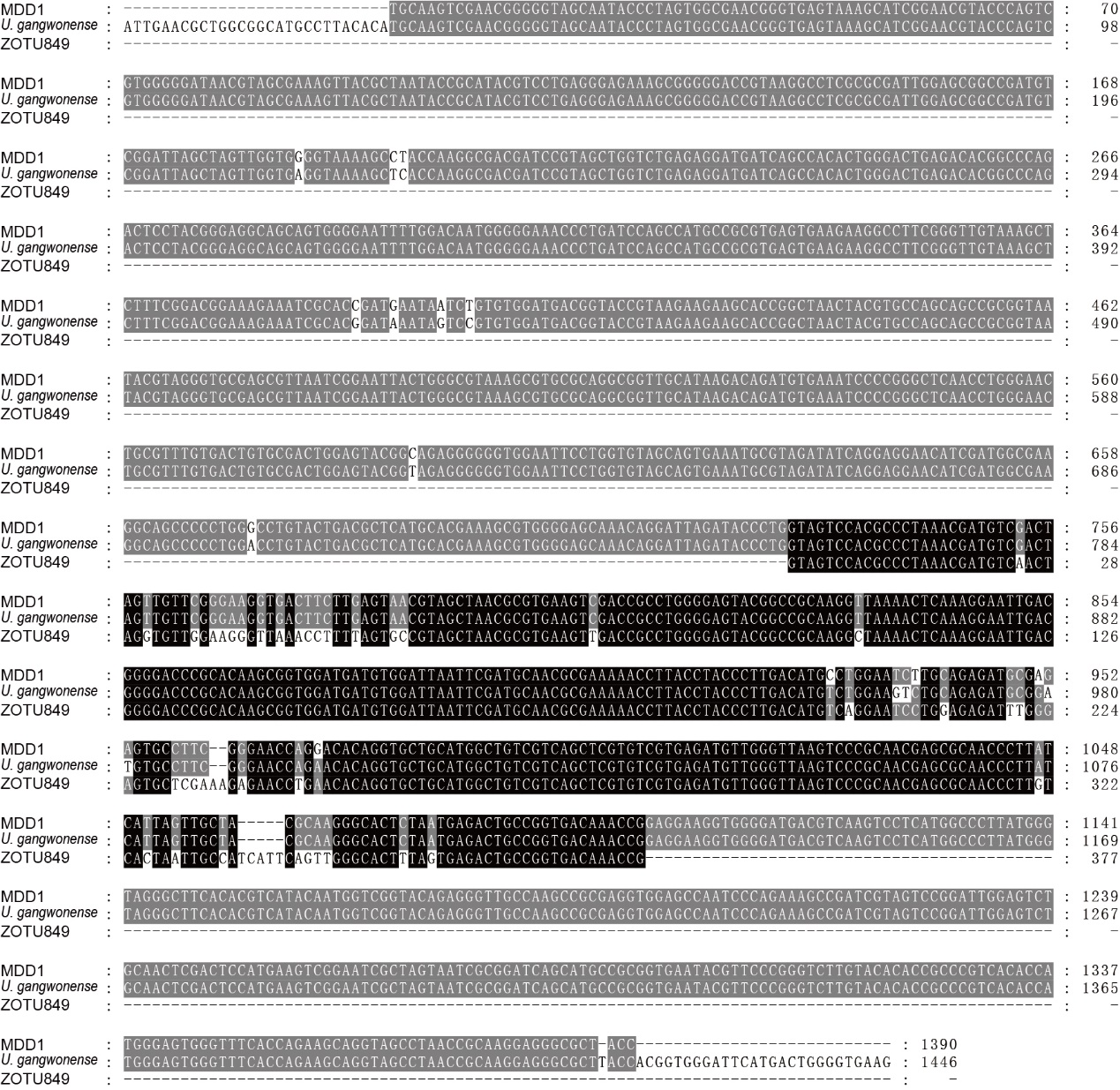


**Figure S11.** Sequence alignments of the 16S rRNA gene of MDD1, *U. gangwonense* strain NBRC 106428, and ZOTU849.


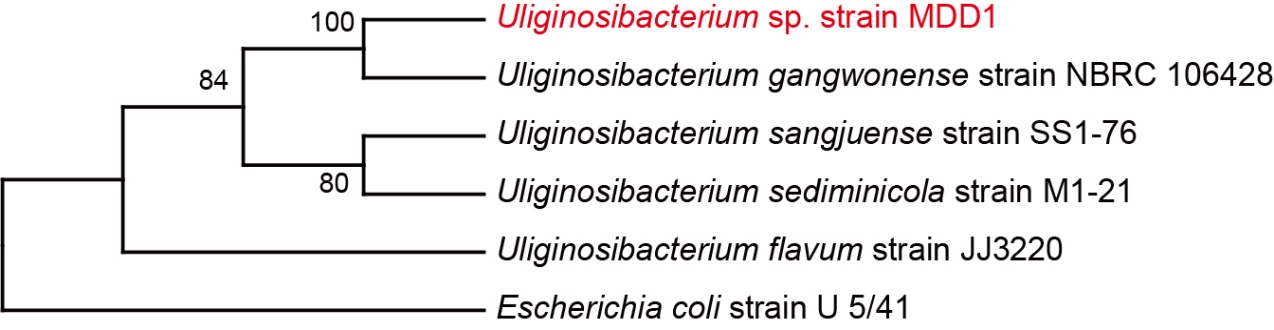


**Figure S12.** Phylogenetic tree of MDD1 and related *Uliginosibacterium* strains based on 16S rRNA genes. The tree was constructed using the Neighbor-joining (NJ) method in MEGA. Numbers at nodes indicate bootstrap support values for 1000 replicates. *E. coli* was used as outgroup. The accession numbers of the 16S rRNA gene sequences used in this phylogenetic analysis are as follows: *Uliginosibacterium gangwonense* strain NBRC 106428, AB682440.1; *Uliginosibacterium sangjuense* strain SS1-76, KU341405.1; *Uliginosibacterium sediminicola* strain M1-21, NR_159920.1; *Uliginosibacterium flavum* strain JJ3220, NR_178617.1; *Escherichia coli* strain U 5/41, ON799228.1.


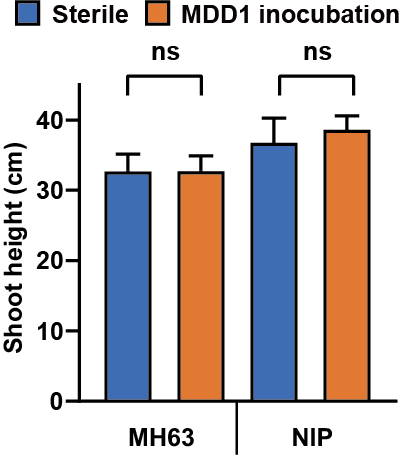


**Figure S13.** Comparisons of shoot height of intact rice seedlings after MDD1 inoculation. ns, no significant difference (student’s *t* test, n = 16); error bar, standard deviation.
